# Proposal and Evaluation of BLE Discovery Process Based on New Features of Bluetooth 5.0

**DOI:** 10.3390/s17091988

**Published:** 2017-08-30

**Authors:** Ángela Hernández-Solana, David Perez-Diaz-de-Cerio, Antonio Valdovinos, Jose Luis Valenzuela

**Affiliations:** 1 Aragon Institute for Engineering Research (I3A), University of Zaragoza, 50018 Zaragoza, Spain; toni@unizar.es; 2 Signal Theory and Communications Department, Universitat Politècnica de Catalunya, Esteve Terrades 7, 08860 Castelldefels, Spain; dperez@tsc.upc.edu (D.P.-D.-d.-C.); valens@tsc.upc.edu (J.L.V.)

**Keywords:** Internet of Things (IoT), BLE, neighbor discovery, non-detection probability, discovery latency

## Abstract

The device discovery process is one of the most crucial aspects in real deployments of sensor networks. Recently, several works have analyzed the topic of Bluetooth Low Energy (BLE) device discovery through analytical or simulation models limited to version 4.x. Non-connectable and non-scannable undirected advertising has been shown to be a reliable alternative for discovering a high number of devices in a relatively short time period. However, new features of Bluetooth 5.0 allow us to define a variant on the device discovery process, based on BLE scannable undirected advertising events, which results in higher discovering capacities and also lower power consumption. In order to characterize this new device discovery process, we experimentally model the real device behavior of BLE scannable undirected advertising events. Non-detection packet probability, discovery probability, and discovery latency for a varying number of devices and parameters are compared by simulations and experimental measurements. We demonstrate that our proposal outperforms previous works, diminishing the discovery time and increasing the potential user device density. A mathematical model is also developed in order to easily obtain a measure of the potential capacity in high density scenarios.

## 1. Introduction

Wireless communications have been used for more than 30 years to provide secure and cost-effective connectivity for data networking, industrial automation, motion control, remote monitoring and other applications. However, new challenges are emerging in the era of the IoT [1]. The number of devices interacting with each other is increasing, while wireless connectivity standards involved in the IoT paradigm (typically short-range, low-power wireless technologies such as Bluetooth, 802.15.4/ZigBee, 802.15.4/6LoWPAN, IEEE 802.11 wireless-local-area-network (WLAN) standards and proprietary technologies) are continually evolving to provide more reliability and power efficiency. At its origins (1998), Bluetooth, was designed with the aim of reducing the wiring of Personal Area Networks (PAN) and quickly became a wireless global standard, to the point that it is the first technology that usually comes to mind when talking about headsets and hands-free kits. However, since version 4.0, with the introduction of BLE, Bluetooth has turned into an ultra-low power wireless technology suitable to be used within the IoT scenario. Nowadays, it is considered an attractive technology for a wide range of applications, including smarthealth, sport and fitness applications, domotics, home electronics, security, intelligent transportation systems, etc. [2,3,4,5,6]. With Bluetooth version 5.0 published last December, the Bluetooth SIG reaffirmed its position within the competitive scenario of IoT. The new specification quadruples range, doubles speed, and increases data broadcasting capacity by 800% of BLE [7]. 

BLE allows the reduction of consumed energy through a fast neighbor discovery process and periodic sleep during connections. An increasing number of researchers have started paying attention to BLE, with BLE 4.0 being the topic of numerous studies. For example, in [8], the authors characterize, both analytically and experimentally, the performance and tradeoffs of BLE as a technology for opportunistic sensor data collection. They developed analytical current consumption and sensor node lifetime models, derived from the behavior of a real BLE platform, and collected data models. In [9], based on experimental results involving 32 BLE devices, the authors investigate the influence of mutual interference on the energy consumption and latency in BLE devices. Given that a relevant issue of many services, and some particular applications, is to ensure that all the devices involved are discovered, many recent studies focus on the discovery mechanism, and on minimizing the discovery time. In fact, advertising is one of the most important procedures of BLE. Understanding how it really works can help to lower the power consumption, improve reliability and speed up the creation of connections and discovery of devices. The topic has been investigated through experimental, simulation and analytical modeling, involving studies focusing on scannable undirected or non-connectable and non-scannable advertising events. For the sake of brevity, from now on we will refer to the non-connectable and non-scannable advertising events just as non-connectable advertising events. In [10], initial and default parameter settings are analyzed in order to obtain a best tradeoff between discovery latency and energy consumption according to various BLE applications for non-connectable advertisements. The authors in [10] also include an analytical model for these quantities (latency and energy consumption) that is applicable to several parameter settings, but assuming a particular scenario where *M* independent pairs of scanners and advertisers are in proximity to each other. In a similar way, Cho et al. in [11,12] develop analytical models and carry out intensive simulations to investigate discovery probability and the influence of various parameter settings on the discovery latency and the energy performance, in this case involving scannable undirected advertising events. The study in [12] involves three scenarios, with one advertiser that is discovered by *N* scanners, *M* advertisers to be discovered by one scanner, and *M* advertisers under *N* scanner coverages, although the analysis is limited to 10 BLE devices and ideal assumptions about BLE implementation are made. 

So, it is clear that BLE discovering capacities and latency become crucial, and it is necessary to evaluate their performance. The increasing amount of literature on the topic reflects this point. This issue becomes especially challenging when a large number of users/devices have to be detected in a short time period, such as sporting events (race tracking, etc.), goods traceability, access control, cattle control, etc., due to frequent access collisions. However, most of the studies, particularly those that focus on analytical and simulation analysis, are limited to assumptions that are far away from being applicable for analyzing the performance of high-density networks. On the other hand, analytical and simulation studies do not take into account the non-idealities present in real devices. In [13], we have shown that these non-idealities have a severe impact on discovery capacity. In this paper, we will focus on a comparative evaluation of scannable undirected vs. non-connectable advertisements to be employed in high density networks to provide the location and transmission of information where a large number of devices are involved. 

We have previously addressed BLE discovery capacities in [13], based on non-connectable undirected advertisements available in version 4.x of BLE. The purpose of [13] was to evaluate the capacities of BLE in order to enable reliable discovery and identification of devices in the shortest possible time, in high-density environments, with no additional data exchange, and including the impairments present in real devices. We concluded that non-connectable undirected advertising was a reliable alternative for discovering a high number of devices (up to 200) in a very short time period, even considering the effects of the non-idealities. Scannable undirected advertising events with scan request and response were excluded, due to the expected increase of non-detection probabilities and, thus, the probability that not all devices were detected would grow. We proposed a mathematical model that considered not only the official specifications, but also the singularities found in real devices. The main drawback of the approach is that the advertisers are not aware that they have been discovered by the scanner, because in BLE version 4.x there is no command to inform the host that the request packet (SCAN_REQ PDU) has been received by the advertiser or, alternatively, that the response (SCAN_RSP PDU) has been actually sent by the advertiser. On the other hand, BLE 5.0 introduces new features that allows us to suggest feasible changes on the discovery process based on scannable undirected advertising events with request and response that result on a reduction and improvement of the discovery latency compared with the non-connectable scheme evaluated in [13]. The mechanism reduces radio interference and energy consumption of the devices. None of the previous works take advantage of the fact that, once discovered, the advertiser can interrupt the sending of packets, so that the probability of collision decreases and, with that, the number of devices that can be discovered in a certain time increases. This was not possible with previous versions of BLE, since there was no way for the advertiser to notify the host that it had been discovered (which it knows when it receives the SCAN_REQ PDU). In BLE 5.0 this possibility has been introduced, and is what is modeled and analyzed by simulation for the first time in this work. The analysis is not limited to the theoretical and ideal processes as described in the standard, and which are the basis of the work of other authors. We have carried out an exhaustive process of experimental measures to characterize the actual operation of the devices. In [13], we did this for the case of non-connectable and non-scannable undirected advertising events, whereas in this article we present the results of characterization of scannable undirected advertising events, which has given rise to a new mathematical model, which closely meets scannable undirected advertising event particularities of real devices, and was developed in order to easily obtain a measure of the potential capacity in dense scenarios. Discovery probabilities and latencies for a varying number of devices and parameters, including the effects of the backoff mechanism, are compared by simulations and experimental measurements. We demonstrate that our proposal outperforms previous works, diminishing the discovery time and increasing the potential user device density.

We have structured the paper in the following way: first we present a brief BLE overview focusing on scannable undirected advertising events and the new discovery procedure proposal. Next, we characterize this mechanism in real devices and infer a state diagram for the main types of scanners analyzed. In Section 4, we develop the analytical model which can be used to study the behavior of the system for different parameters. Subsequently, we present and discuss the experimental, simulation and analytical results in Section 5. Finally, in Section 6, we extract and summarize the main conclusions observed from the obtained results.

## 2. BLE Overview and Discovery Procedure Proposal

Bluetooth has evolved through five main versions; all versions of the Bluetooth standard maintain downward compatibility. In this paper, we focus on discovering, with the minimum possible delay, the devices located in a predefined scenario. The communications considered are connection-less, using the advertising mechanisms defined in the BLE specifications. However, instead of using non-connectable and non-scannable undirected advertising events, the proposal is based on scannable undirected advertising events. As we will show in the next section, this procedure generates more packets and, therefore, more interference. Nevertheless, the latest version, Bluetooth 5.0, introduces new functionalities. The aim is to take advantage of one of these improvements, the new LE Scan Request Received event. This event indicates that a SCAN_REQ PDU or an AUX_SCAN_REQ PDU has been received by the advertiser. By using the LE Scan Request Received event, we can suspend temporally the transmission of advertising events, reducing considerably the collision probability and energy consumption.

In order to fully understand the operation of the system, next we briefly summarize the broadcasting procedure and the interchange of involved packets, as well as their structure. Finally, we introduce the main assumptions linked to the proposal.

### 2.1. Overview of Scannable Undirected Advertising Events

**Figure 1:**
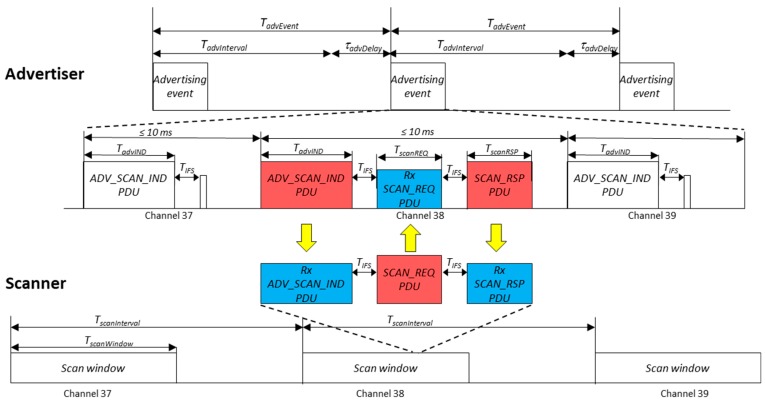
Example of a scannable undirected advertising event.

As stated before, in this study we use scannable undirected advertising events. Basically, in this procedure, a device configured in advertising mode, named advertiser, periodically initiates advertising events in order to be discovered and send information. For every advertising event, the advertiser broadcasts advertising information (ADV_SCAN_IND PDU) in sequence over each of the three advertising channels (index = 37, 38 and 39). Although this is the behavior by default, this channel mask can be modified to use any combination of these three channels. When an ADV_SCAN_IND packet is received by a device configured in *active scanning* mode, the scanner is allowed to demand more information using a scan request (SCAN_REQ PDU). If applied, this packet is sent 150 μs (*T_IFS_*) after the successful reception of the ADV_SCAN_IND. When the advertiser receives the scan request packet, it checks if the scanner address is in its white list filter, if applicable. In this case, it responds with the corresponding scan response, a $T_{IFS}$, later on the same channel. The advertising event is repeated after a $T_{advEvent}$, which corresponds to the sum of a fixed interval ($T_{advInterval}$) and a random delay ($\tau_{advDelay}$), to avoid collisions. $T_{advInterval}$ shall be an integer multiple of 0.625 ms in the range of 20 ms to 10,485.759375 s; and $\tau_{advDelay}$ is a pseudo-random value with a range of 0 ms to 10 ms. Periods between ADV_SCAN_IND packets shall be less than 10 ms. The visual representation of this procedure is shown in Figure 1.

**Figure 2:**
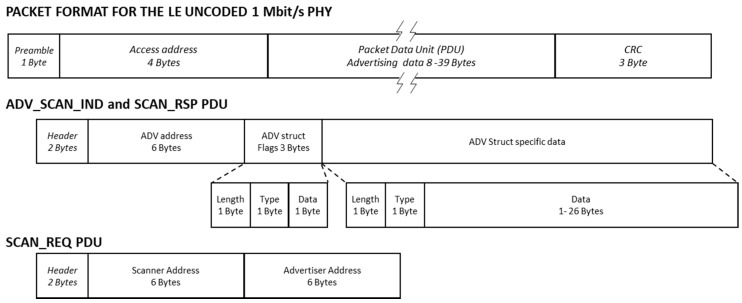
Packet formats present in a scannable undirected advertising event.

Figure 2 depicts the structure of the different packets involved in a scannable undirected advertising event. Throughout the paper, we will use varying data content for the ADV_SCAN_IND and SCAN_RSP packet data units (PDU) in order to evaluate a suitable sample of results. The final values employed in each case will be defined when needed.

Additionally, the standard states that the scanner shall minimize the collision of scan requests packets in a scenario with several scanners using a backoff procedure. Although this fact is mandatory, the standard only proposes an example of such a procedure. When two or more scanners collide, the algorithm proposed restricts the transmission of scan request packets based on two variables, *backoffCount* and *upperLimit*. When the device enters the scanning state, both variables are set to one. Then, on every received ADV_SCAN_IND allowed by the scanner filter policy, the *backoffCount* is reduced by one. When this value reaches zero, the scan request is transmitted. After sending a scan request, the scanner listens for a scan response coming from the expected advertiser. If a valid scan response is received, it is assumed to have been a success; otherwise it is assumed to have been a failure. When there are two consecutive errors, the *upperLimit* is duplicated until a maximum value of 256. On the other hand, when two valid and consecutive scan responses are received, the *upperLimit* is divided by two until the minimum value of one. Every success or failure, the scanner selects a pseudo-random value for the *backoffCount* between one and *upperLimit*.

### 2.2. Adapted Discovery Process

As we anticipated above, the specification v5.0 defines the LE Scan REQ Received event, which indicates to the upper layer of the advertiser that a SCAN_REQ PDU has been received. This introduces the possibility that the advertiser stops the advertising process. After receiving a valid scan request, the advertiser may assume that it has been discovered. The advertiser shall reply with a scan response, but no matter whether the reception of the SCAN_RSP PDU was successful or unsuccessful, the advertising process may be ended, the fact that it may be resumed after a configured period of time notwithstanding. Note that, in relation to the potential applications that we are interested in, the advertisers are required to be discovered at least once, but are not required to be discovered more than one time, and by no more than one scanning device in a coverage area. Thus, continuous advertising events spaced by advertising intervals are not required. It is true that, after that, the advertiser may be required to wake up in order to be detected in subsequent coverage regions. However, potential triggers and parameter configuration to control the wake-up process in practical applications are beyond the scope of this work. In a first phase, the focus is on qualifying the discovery capacities in dense BLE scenarios where a large number of devices need to be discovered in a short time period. 

In contrast to the non-connectable scheme with only advertising PDUs previously characterized in [13], scannable undirected advertising events with SCAN_REQ and SCAN_RSP PDUs allow the advertiser to know if it has been discovered by the scanner after successful detection of the SCAN_REQ. Nevertheless, if continuous advertising events are configured, the advertisers keep on sending a new ADV_SCAN_IND PDU every advertising interval. Collisions between BLE devices grow due to the higher number of signaling packets sent in the radio channel (SCAN_REQ and SCAN_RSP PDU transmissions). As a result, non-detection probabilities increase, and the probability of not detecting all the present devices within a window of opportunity grows. This may challenge the applicability of the solution. On the contrary, stopping the advertising process after the first SCAN_REQ detection not only avoids unnecessary energy waste, but also reduces the time required to detect all BLE devices. Thanks to this modification in the discovery procedure, we will demonstrate that very significant improvements are obtained with respect to the previous proposals in terms of the mean detection time and the detection probability of all the devices in a given time. In addition, the analysis has been performed for a large number of advertisers, when the effects of packet collisions are more pronounced, as the ADV_SCAN_IND PDU sent by an advertiser may collide with other ADV_SCAN_IND PDUs sent by other advertisers, as well as with the SCAN_RSP PDU sent by a recently discovered advertiser, or with the SCAN_REQ PDU sent by the scanner upon successful reception of an ADV_SCAN_IND PDU. On the other hand, the BLE specification defines that the scanner shall use a backoff procedure. This procedure can have a severe impact on the discovery capacities in a dense BLE scenario, such as the one considered here, even though only one scanner is present. The specification does not define a specific implementation, only suggesting an example of implementation. Thus, differences between manufacturers may be significant, as we will show in Section 3. In any case, it seems clear that if, as suggested in the scheme proposed by the specification, the failure on receiving an expected SCAN_RSP PDU from an advertiser is used to control the backoff process, the discovery capacity may result severely and unnecessarily degraded. The use of non-detection of the SCAN_RSP PDUs as an indication of SCAN_REQ collisions between scanners will typically be wrong in a highly dense scenario, where we often have non-detections of SCAN_RSP due to collisions of transmitted SCAN_RSP with ADV_SCAN_IND sent by other advertisers in the coverage area. In this work, the importance of the backoff procedure carried out by the scanners has been demonstrated and quantified. Throughout the tests, we detected that some of the BLE device manufacturers implement the backoff algorithm suggested by the standard, and other manufacturers do not. As one of the key points of this work is the characterization and modeling of real devices, and as the backoff has great impact in the device discovery process, we have included these two options in our study. Nevertheless, the backoff in BLE is a subject not sufficiently studied [14,15], and other backoff procedures should be further investigated in depth. The authors in [14] propose an algorithm that eliminates the fixed synchronization of 150 μs existing in the standard between the ADV_SCAN_IND, SCAN_REQ and SCAN_RSP packets, and introduce a random response time for the sending of the SCAN_REQ PDU by the scanner. In [15], a randomization of the frequency scanning sequence of each scanner is proposed, so that if two scanners coincide in the scan frequency and collide their SCAN_REQ PDUs, the probability of collision in the subsequent transmission decreases by following different sequences in the frequencies that they scan. The problem of both proposals for practical implementation is that they are not compatible with the current versions of the Bluetooth standard. Since the implementation of the backoff algorithm may be very different between manufacturers, and as it is a challenging issue that needs to be further studied, it has not been included in the analytical models we present in Section 4. Backoff effects will be evaluated only by simulations, according with the implementation suggested in the standard.

## 3. Characterization of the Scannable Undirected Advertising Mechanism in Real Devices

**Figure 3:**
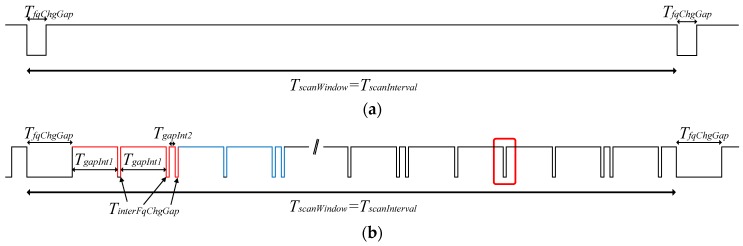
Blind times due to non-idealities of real devices. (**a**) Type 1 scanners; (**b**) Type 2 scanners.

In [13], we characterized the neighbor discovery process based on non-connectable advertising events, with only ADV_NONCONN_IND PDUs, and we demonstrated the impact of the impairments of real devices. We measured the behavior of different chipset manufacturers. All scanning devices present undesired pauses in the scanning (blind times), increasing the non-detection probability. These pauses appear even when we consider just one scanner without any advertiser present. When continuous scan behavior is configured ($T_{scanWindow}=T_{scanInterval}$), all chipset manufacturers follow, with slight variations, two behavior patterns that we identified in [13] as types 1 and 2. Figure 3 summarizes the effects of the non-idealities analyzed and discussed in [13]. In both types, a gap appears when the scanner changes the scanning frequency and its duration is $T_{fqChgGap}$. In addition to frequency change gaps, in type 2 scanning devices there are also other periodic short pauses with duration $T_{interFqChgGap}$. These gaps appear following a periodic pattern, having $T_{gapInt1}$ and $T_{gapInt2}$ as its characteristic variables. 

Besides these pauses, the scanner has an additional blind time whenever a packet is received. These pauses are associated with the received or expected packet processing time, and we have named them *decoding gaps*. These gaps should not be ignored, because if another packet arrives during this blind time, it will not be detected.

Now, the scannable undirected advertising mechanism is quite different from the non-connectable undirected advertising studied in our previous work. Bidirectional transmission, collision increase and interference must be analyzed. On the other hand, the backoff algorithm needs to be characterized. We designed physical and MAC layer experimental measurements in order to understand the real behavior of BLE devices and to obtain an accurate characterization. This characterization allows us to extend the analysis for a high number of devices and several parameter settings using simulations and, additionally, to obtain an analytical model. Section 3.1 focuses on receiver measurements, which describe the real receiver baseband and MAC state characteristics of the Bluetooth devices, described in Section 3.2.

### 3.1. Measurement Setup Description

**Figure 4:**
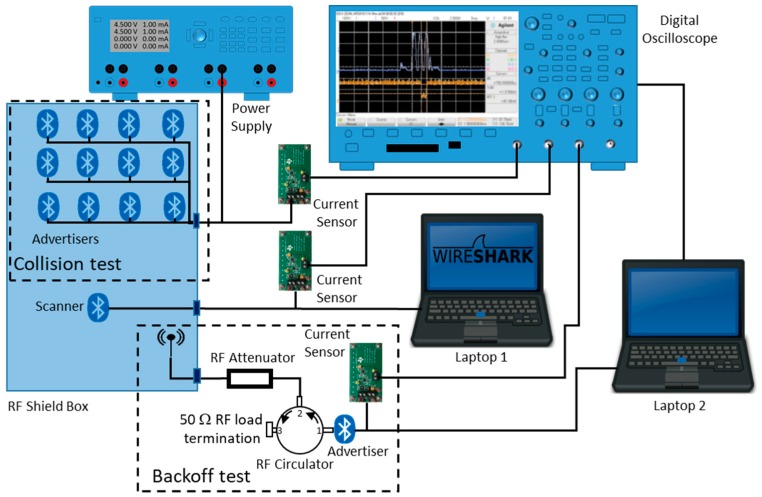
Experimental test-bed.

We performed three main tests in the scenario using the schema represented in Figure 4. First of all, we designed a collision test. In this case, we placed a scanner and up to 18 advertisers inside an RF-shield box. A laptop was employed to control the scanner and capture the Bluetooth Host Controller Interface (HCI) data using *Tshark* [16]. With this configuration, we fixed $T_{scanWindow}$ and $T_{scanInterval}$ to 500 ms to maintain a continuous active scanning. The advertisers were configured with the following parameters: advertising interval ($T_{advInterval}$), size of the advertising data ($T_{advIND}$) and size of the scan response data ($T_{scanRSP}$). The parameter values were set according to the evaluation conditions defined in Section 5. The experiment duration was 180 min for capturing packets with each of the different configurations. Then, we processed the raw data and calculated the non-detection probability of advertising and scan response packets and the time between consecutive detections among other statistics. Results will be presented later, combined with the ones of the analytical model and simulations. 

Secondly, we designed a similar configuration to analyze the receiver behavior when it receives scannable undirected advertising events. This is because when a packet is received, the scanner momentarily abandons the scanning state to process the packet; producing, in this way, different pauses from those already analyzed. We characterized the behavior of the devices by simultaneously monitoring in an oscilloscope the instantaneous current consumption of the advertisers and the scanner using current sensors, the design of which was based on [17]. As in [13], the aim was to analyze the current consumption of the devices to extract behavior patterns of the scanner when it is receiving scannable undirected advertising events. However, in this case, we combined the information obtained by behavior patterns with those obtained with *Tshark*. Thus, we were able to obtain information about synchronization, packet detection, collision between ADV_SCAN_IND, SCAN_REQ and SCAN_RSP packets, capture effects, etc. We processed the combined *Tshark* and the oscilloscope data in order to infer a receiver state diagram.

Finally, we conceived a configuration in order to analyze the backoff algorithm implemented in the scanner. The proposed backoff procedure of the specifications was designed to reduce the collisions between several scanners, as explained in Section 2.1. However, this procedure could be also activated with a single scanner. This happens when the transmitted SCAN_REQ or the SCAN_RSP packets are not received by either the advertiser or the scanner because they collide with ADV_SCAN_IND packets from other devices, or are not detected correctly.

To cause this effect, we used the setup marked as backoff test in Figure 4. The packets generated by the advertiser are transmitted through a circulator and an attenuator. When the scanner detects the ADV_SCAN_IND packet, it responds with a SCAN_REQ packet. This packet is not received by the advertiser because the signal applied to port 2 of the circulator only comes out of port 3. Then, the advertiser does not send the SCAN_RSP and the scanner activates the backoff algorithm.

### 3.2. Active Scanning State Diagrams

With the combination of the results of the three tests developed over last section, we inferred a state diagram for the two different types of scanners.

#### 3.2.1. Type 1 Scanner State Diagram

**Figure 5:**
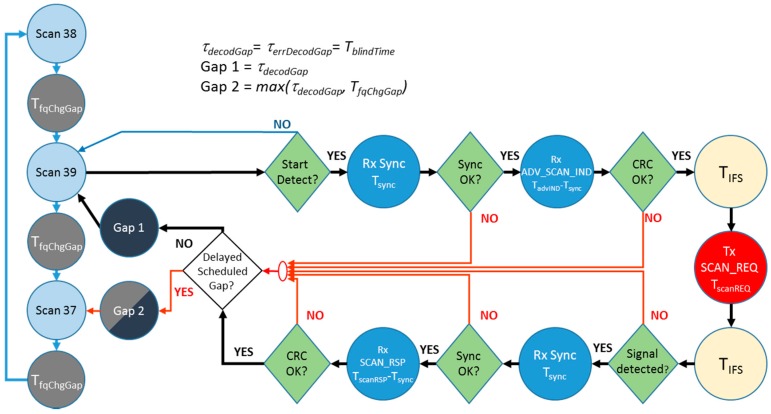
Type 1 scanner state diagram.

Figure 5 depicts the state diagram for the first type of scanner characterized. On the left, and distributed vertically, we see the cyclic procedure of scanning the three different advertising channels (37 → 38 → 39 → 37 …) with its corresponding frequency change blind time of 1.1 ms ($T_{fqChgGap}$) between each state, which corresponds with the behavior represented in Figure 3a.

In this figure, the diagram supposes that the device under test (DUT) was scanning on channel 39; nevertheless, the behavior is the same for any of the other frequencies. The scanner remains in this state until the start of a packet is detected. When this happens, the scanner tries to synchronize during ($T_{sync}$) with the possible received advertisement.

In the case of synchronization failure, the scanner aborts the packet processing procedure and enters into a blind time. We named this a *errDecodGap*, similar to *decodGap*, as defined in [13], the duration of which ($\tau_{errDecodGap}$) is a uniform distribution between $T_{minErrDecodGap}$ and $T_{\max ErrDecodGap}$ whose values are 350 μs and 1.6 ms, respectively. The reasons behind this failure are a nearly perfect overlap with another packet, or the reception of a ADV_SCAN_IND while there a previous packet is still active from another device that did not initiate the decoding process. The receiver always tries to process the first packet received when coming from the scanning state. If the process has already been initiated when another packet is received, we confirmed that this second packet would always be discarded. If the synchronization is successful, the scanner waits for the complete reception of the ADV_SCAN_IND and checks its CRC. The CRC results in a failure in case of poor channel conditions or if the ADV_SCAN_IND collides with another PDU (ADV_SCAN_IND or ADV_RSP). In this case, an *errDecodGap* is introduced.

When the CRC check is passed, the scanner initiates the process of sending a SCAN_REQ. It waits for a $T_{IFS}$, sends the SCAN_REQ, which has a duration of 176 μs, and waits for another $T_{IFS}$ before listening for the SCAN_RSP. If it does not detect any signal, it generates another blind time, with the same duration of the *errDecodGap*. On the contrary, it tries to synchronize with the received SCAN_RSP and checks its CRC in a similar way as done with the ADV_SCAN_IND. In this case, the scanner makes an *errDecodGap* when there is a failure on the synchronization. If the synchronization is successful, it also introduces a *decodGap* after the CRC check no matter if it is successful or not. When successful, *decodGap* ($\tau_{decodGap}$) follows the same uniform distribution of $\tau_{errDecodGap}$. When the CRC is successful, the scanner generates two HCI report events to the upper layer with the contents of the ADV_SCAN_IND and SCAN_RSP received. In case of failure, the report only includes the ADV_SCAN_IND.

As we have seen, the *decodGap/errDecodGap* is always introduced before returning to the scanning state once the processing of a packet has been initiated. If a frequency change is scheduled within this process, it will be postponed until the start of the *decodGap/errDecodGap.* In this case, if this *decodGap/errDecodGap* and also the postponed $T_{fqChgGap}$ occur simultaneously, the scanner only applies the largest of them.

Another important fact regarding this type of device is that we have verified that they do not implement a backoff algorithm, although it is mandatory in the standard.

#### 3.2.2. Type 2 Scanner State Diagram

**Figure 6:**
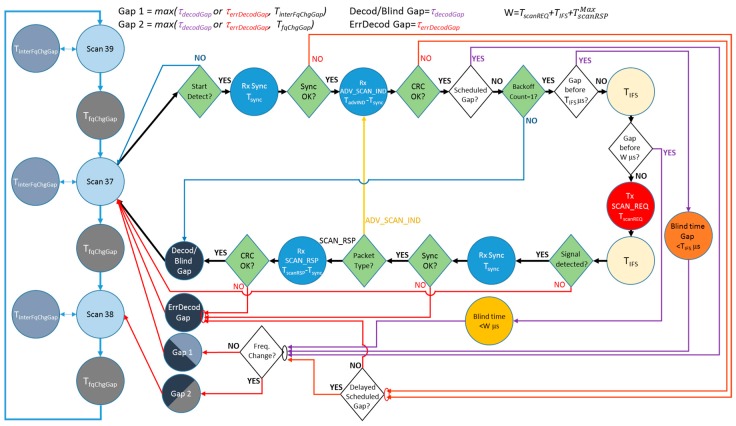
Type 2 scanner state diagram.

Figure 6 depicts the state diagram for the second type of scanner characterized. In comparison with the state diagram for type 1 scanners, the state diagram in this case is somehow more complex.

The basic operation is similar; the scanner cycles over the three different frequencies in a round-robin fashion with a small blind time between them ($T_{fqChgGap}$). In this case, this value is greater than before, at 16.05 ms. 

Additionally, to reproduce the behavior shown in Figure 3b, the scanner may exit now from the scanning state to introduce several $T_{interFqChgGap}$ gaps, periodically. The details and specific values for this behavior are described thoroughly in [13].

In a similar way to type 1 scanners, while the device is in any of the scanning states, once it starts detecting energy on the channel, it begins packet processing. However, unlike the previous case, now, when there is a failure in the synchronization or in the CRC check, the introduced gap will be constant and considerably shorter than before ($\tau_{errDecodGap}$ is 144 μs). Moreover, before returning to the scanning state, it is necessary to consider whether there was a postponed periodic gap (named a scheduled gap). In this case, the scheduled gaps may be not only the $T_{fqChgGap}$, but also the $T_{interFqChgGap}$.

Another difference between the two device types is that, after a successful CRC check, type 2 devices apply the backoff algorithm described in Section 2.1. If the *backoffCount* is greater than one and, therefore, the SCAN_REQ is not sent, the scanner returns to the scanning state after introducing a blind time equal to the *decodGap*, with $\tau_{decodGap}$ being constant and equal to 194 μs. In this case, an HCI report event with the contents of the ADV_SCAN_IND is generated to the upper layer. In contrast to Type 1, if a SCAN_REQ is to be transmitted, the device first checks if there is a periodic gap ($T_{fqChgGap}$ or $T_{interFqChgGap}$) scheduled before the completion of the process. In these cases, the transmission of the SCAN_REQ is aborted. If a periodic gap is expected to be scheduled before a $T_{IFS}$, the scanner remains in a blind state for as much time as remains for the scheduled periodic gap. Finally, if a scheduled periodic gap was programmed between the end of the $T_{IFS}$ and before the expected complete reception of the SCAN_RSP, the scanning device enters a blind time (waiting state) until the scheduled instant, and then introduces the periodic gap. From the point of view of the scanner, the expected duration of the SCAN_RSP will be the maximum allowed ($T_{scanRSP}^{MAX}$); thus, the waiting time has a duration of up to $T_{scanREQ}+T_{IFS}+T_{scanRSP}^{MAX}$.

Finally, when the SCAN_REQ is transmitted after a $T_{IFS}$, the scanner waits for the SCAN_RSP. If the synchronization is correct, an additional check is done to verify the packet type. If the received packet is another ADV_SCAN_IND, it returns to the point to check the CRC of the ADV_SCAN_IND. However, if the packet is the awaited SCAN_RSP, it checks its CRC. If this is successful, the scanning device introduces a *decodGap* and generates the corresponding two HCI report events to the upper layer, one for the ADV_SCAN_IND and one for the SCAN_RSP. If not, it only generates an HCI report event for the ADV_SCAN_IND and introduces an *errDecodGap*.

## 4. Analytical Model

In this section, we describe the mathematical model that allows us to characterize the BLE device discovery process. The model is derived according with the Bluetooth standard 5.0, but including the peculiarities of different implementations performed by the chipset manufacturers. We narrow our focus to deriving the performance metrics of the proposed interrupted version of the scannable undirected advertising event. This objective implies a previous characterization of the standard implementation of this same scheme without interruption. The final purpose is to compare both continuous and interrupted versions of the scannable undirected advertising event with the non-connectable event with only advertising PDUs (previously studied in [13]).

The mathematical models developed here will be a useful instrument for effortlessly calculating the upper bounds of the discovery capacity, and for choosing the values of the parameter settings that control the advertising process, according to a particular BLE application. The two main configurations have their own peculiarities that prevent them from using the same quantities, but there is a set of parameters that allows the main capacities to be derived, and a fair comparison to be performed. The analytical models allow the characterization of the following parameters:

- Non-detection probabilities of ADV_SCAN_IND, SCAN_REQ and SCAN_RSP.
- Mean discovery latency, associated with two possible parameters:
  ○ Average ADV detection delay, defined as the time interval between the instant a BLE device enters advertising mode and the time instant when the ADV_SCAN_IND is successfully received by the scanner.
  ○ Average SCAN_REQ detection delay, defined as the time interval between the instant a BLE device enters advertising mode and the time instant when the SCAN_REQ is successfully received by the advertiser.
- Average time required for discovering all devices, defined as the time required for detecting all the BLE devices in the coverage area.
- Probability that not all the BLE devices present in the scanner coverage area will be detected within a limited time interval (window of opportunity or dwell time).

These parameters are in addition to:

- The mean time between consecutive ADV_SCAN_IND, SCAN_REQ or SCAN_RSP successful detections, associated to an advertising device.
- The mean number of ADV_SCAN_IND, SCAN_REQ or SCAN_RSP successful detections within a window of opportunity.

The mathematical characterization starts from the calculus of the collision probability between ADV_SCAN_IND PDUs, assuming the ideal operation of BLE, in accordance with the standard (denoted as $P_{NDAdvIND}^{col}$). Afterwards, we will employ it to obtain the overall ADV_SCAN_IND non-detection probability (denoted as $P_{NDAdvIND}^{}$). In this case, the impairments of real BLE chipset implementations are included in the $P_{NDAdvIND}^{}$ derivation, in accordance with characterizations performed in Section 3. $P_{NDAdvIND}^{}$ will depend on several components: the collisions between ADV_SCAN_IND packets from different advertisers, non-detections due to the scanner being involved in the exchange of the following control messages (SCAN_REQ, SCAN_RSP) associated with the scanning procedure of another advertiser whose ADV_SCAN_IND has been successfully detected, preplanned *scanning gaps* identified in Section 3, post-processing *decoding gaps* and BLER (Block Error Rate) due to interference, and noise and channel conditions. Subsequently, we calculate the SCAN_REQ and SCAN_RSP non-detection probabilities, which in turn will condition the length of the time periods in which the scanner is involved in the exchange of control messages during the scanning procedure. Consequently, they condition the probability of not detecting an ADV_SCAN_IND. The interrelation between the involved variables implies that the applied solution is iterative in several stages of the analytical model.

Given the similarities between the ideal and type 1 scanning devices, we first model the non-detection probabilities for these devices. Next, we include some variations to characterize the type 2 scanning devices. Then, we obtain the main performance parameters used on the evaluation as the average time required to discover all the devices under the scanner coverage area. Finally, in Section 5, we will prove that the proposed mathematical model closely meets both the experimental and simulation results obtained for a wide range of variation in the number of coexisting BLE advertising devices.

The mathematical model used to obtain the non-detection probabilities of the ADV_SCAN_IND, SCAN_REQ and SCAN_RSP is based on parameters and variables summarized in Table 1 and Table 2, in a scenario with $N_{BLE}$ advertisers. In order to simplify the notation, dependence on the number of advertisers present in the scenario is not included in the notation.

As general considerations, we assume that $N_{BLE}+1$ devices are present in the scenario: a scanner device located in a fixed position plus $N_{BLE}$ advertisers that remain in coverage of the scanner during a certain time period. As the objective is to discover the presence of a large number of devices in a short time period, the scanner is configured to scan 100% of the time; that is, $T_{scanInterval}=T_{scanWindow}$_._ A collision occurs when the PDU transmissions (ADV_SCAN_IND, SCAN_REQ or SCAN_RSP) of at least two devices (scanner or advertisers) are time-overlapped on the same frequency channel. We assume that interference conditions are the same in the three available channels (37, 38, 39), and that all the advertiser devices are configured with the same parameter settings. Then, without loss of generality, we can characterize the non-detection probabilities assuming that both the scanner and the advertisers are always scanning and transmitting, respectively, at the same frequency.

To derive the analytical model, the same assumption can be made for ideal and real devices: the starting time of the advertising event for a device in each channel is independent of each other device, and is not affected by collisions or non-detections throughout the overall discovery process. Therefore, we can firstly obtain three preliminary non-detection probabilities that we will use as a basis for the analytical models.

The collision probability between ADV_SCAN_IND PDUs in a scenario with $N_{BLE}$ advertisers is obtained with Equation (1). Note that, when setting a reference advertiser whose transmission starts at time instant *t*, a collision occurs with any other that initiates its transmission in the time interval $\left[t-T_{advIND},\;t+T_{advIND}\right]$. Given the time interval between consecutive ADV_SCAN_IND transmissions $T_{advInterval}+\bar{\tau_{advDelay}}$, the collision probability between two devices is $2\cdot T_{advIND}/(T_{advInterval}+\bar{\tau_{advDelay}})$. Transmissions of $N_{BLE}$ devices are independent; thus, the probability that the reference device collides with any of the other $N_{BLE}-1$ devices is one minus the probability of not colliding with any of them. Note that collisions between ADV_SCAN_IND and SCAN_REQ or SCAN_RSP are not included in this variable.

(1) $$P_{NDAdvIND}^{col}=1-{\left(1-\frac{2\cdot T_{advIND}}{\bar{T_{advEvent}}}\right)}^{N_{BLE}-1}\;\mathrm{with}\;\bar{T_{advEvent}}=T_{advInterval}+\bar{\tau_{advDelay}}$$

Once an ADV_SCAN_IND is detected by a scanner, the scanner is allowed to transmit a scan request to obtain additional information. In this case, the probability that the SCAN_REQ transmission (started in a time instant *t*) is not detected by the advertiser due to collision with an ADV_SCAN_IND transmission from one of its neighbor devices depends on the probability that the ADV_SCAN_IND transmission of another device starts in the time interval $\left[t-\min(T_{IFS},T_{advIND}),\;t+T_{scanREQ}\right]$. However, note that a transmission that started in the interval $\left[t-T_{advIND},\;t-T_{IFS}\right]$, given $T_{advIND}>T_{IFS}$, would imply the non-detection of the ADV_SCAN_IND that is supposed to trigger the SCAN_REQ response. Thus, this case is not possible. As $T_{advInterval}+\bar{\tau_{advDelay}}$ is the time interval between advertisements transmissions, the probability of collision is $\left(\min(T_{IFS},T_{advIND})+T_{scanREQ}\right)/\bar{T_{advEvent}}$. In the same way that $P_{NDAdvIND}^{col}$, the probability of collision between a SCAN_REQ and ADV_SCAN_IND transmissions is given by Equation (2):

(2) $$P_{NDScanREQ}^{col}=1-{\left(1-\frac{\min(T_{IFS},T_{advIND})+T_{scanREQ}}{\bar{T_{advEvent}}}\right)}^{N_{BLE}-1}$$

Following analogous considerations, Equation (3) characterizes the non-detection probability of a SCAN_RSP transmission caused by collisions with ADV_SCAN_IND transmissions from any other of its neighbor devices. The non-detection probability of the SCAN_RSP transmission (started in a time instant *t*) due to collision with an ADV_SCAN_IND transmission from one of its neighbor devices depends on the probability that the ADV_SCAN_IND transmission of a neighbor device starts in the interval $\left[t-\min(T_{IFS},T_{advIND}),\;t+T_{scanRSP}\right]$. As $T_{advInterval}+\bar{\tau_{advDelay}}$ is the time interval between transmitted advertisements, the collision probability is $\left(\min(T_{IFS},T_{advIND})+T_{scanRSP}\right)/\bar{T_{advEvent}}$. A transmission that started in the interval $\left[t-T_{advIND},\;t-T_{IFS}\right]$, given $T_{advIND}>T_{IFS}$, would imply the non-detection of the SCAN_REQ that is supposed to trigger the SCAN_RSP. Given that transmissions of the $N_{BLE}$ devices are independent, the SCAN_RSP collision probability with ADV_SCAN_IND transmissions of other devices is one minus the probability of not colliding with any of them.

(3) $$P_{NDScanRSP}^{col}=1-{\left(1-\frac{\min(T_{IFS},T_{advIND})+T_{scanRSP}}{\bar{T_{advEvent}}}\right)}^{N_{BLE}-1}$$

### 4.1. Non-Detection Probabilities for the Ideal and Type 1 Chipsets

Starting from the non-detection probabilities due to collisions included above, in this section we describe a model that provides a complete characterization of the non-detection probabilities. The model includes the particularities of the scanning procedure with SCAN_REQ and SCAN_RSP PDUs, and also the behavior particularities of the manufactured BLE chipsets. In accordance with the characterization performed in Section 3, the non-detection probability is affected by two types of scanning pauses, which are included separately in the model. That is:

- The *periodic scanning gaps*. This kind of gap is always present. $P_{NDAdvIND}^{scanGap}$ denotes the non-detection probability of ADV_SCAN_IND due to these periods.
- The *decoding gaps.* These gaps appear whenever the scanner decodes a packet or is unable to detect an expected SCAN_RSP PDU after a specific timeout. Consequently, it depends on the number of PDUs the scanner is detecting. That is, it really depends on the number of BLE advertisers in the scanner coverage. $P_{NDAdvIND}^{decodGap}$ denotes the non-detection probability of the ADV_SCAN_IND caused by these blind times.

Ideal implementations according to the specification and type 1 real devices can be characterized with the same model, by only giving the value zero to the *periodic scanning gaps* and the *decoding gaps* when the ideal case is considered. The main characteristic that allows this assumption is that *periodic scanning gaps* (which, in this case, are only associated with change frequency gaps) are prevented from interrupting the general process. We have seen that, if an advertising event is initiated and, during the ADV_SCAN_IND reception, the scanner has scheduled a periodic gap, this gap is postponed at least until the reception is finished (if synchronization is correct), regardless of whether the reception is correct or a collision or error occurs. Additionally, if the ADV_SCAN_IND reception is correct, or if the *periodic scanning gap* is planned to start once the ADV_SCAN_IND has been correctly received, the periodic gap is delayed up until the end of the SCAN_RSP reception or until the timeout on the SCAN_RSP reception is reached.

Derived from the $P_{NDAdvIND}^{col}$, $P_{NDScanREQ}^{col}$ and $P_{NDScanRSP}^{col}$ probabilities, we first obtain the overall non-detection probability of an ADV_SCAN_IND transmission ($P_{NDAdvIND}^{}$). We note that, in addition to collisions with other ADV_SCAN_IND ($P_{NDAdvIND}^{col}$), an ADV_SCAN_IND transmission would be unable to be detected if the scanner were involved in the following events:

*a.* A *signaling processing period*. That is, the exchange of the following control messages associated to the discovery procedure of another advertiser: SCAN_REQ, SCAN_RSP. In a scenario with $N_{BLE}$ advertisers, two advertisers cannot simultaneously trigger the exchange of the control messages. However, in the time period between two consecutive advertisements from a “reference” device, the rest of the devices may trigger $N_{BLE}-1$, $N_{BLE}-2$, ..., one or no signaling processing gap on the scanner, depending on the ADV_SCAN_IND non-detection probability. Consequently, we can obtain the mean time that the scanner is involved in a signaling processing period ($\bar{T_{sigproc}}$) within an interval $T_{advInterval}+\bar{\tau_{advDelay}}$ by multiplying the average time of these signaling processing periods ($\bar{\tau_{sigproc}}$) by the average number of devices that may generate it ($\bar{N_{detAdvIND}}$). $\bar{N_{detAdvIND}}$ is obtained according to Equation (4), given the number of neighbor advertising devices $N_{ngdev}=N_{BLE}-1$. The population of advertising devices is finite, so the probability of having *n signaling processing periods* follows a binomial distribution, which depends on the overall non-detection probability of an ADV_SCAN_IND ($P_{NDAdvIND}^{}$). However, at the beginning of the iterative resolution process, $P_{NDAdvIND}^{}$ is initialized by setting $P_{NDAdvIND}^{}=P_{NDAdvIND}^{col}$.

(4) $$\bar{N_{detAdvIND}}=\sum_{n=1}^{N_{ngdev}}n\cdot \left(\begin{array}{l} N_{ngdev}\\ n \end{array}\right){\left(1-P_{NDAdvIND}^{}\right)}^{n}\cdot {\left(P_{NDAdvIND}^{}\right)}^{\left(N_{ngdev}\right)-n}\;\mathrm{with}\;N_{ngdev}=N_{BLE}-1$$

Concerning the duration of the signaling processing period, the time interval needed to exchange control messages always includes an interval $T_{IFS}+T_{ScanREQ}+T_{IFS}$ and a variable time that depends on the successful transmission of the SCAN_REQ PDU (see Equation (5)). If the advertiser receives the SCAN_REQ PDU, it shall reply with a SCAN_RSP, but in the other case, after a timeout (synchronization time) without receiving the expected SCAN_RSP, the scanner moves to a *decoding gap* (type 1 real device) or to the scan mode. The ADV_SCAN_IND non-detection probability due to the *signaling processing periods* ($P_{NDAdvIND}^{sigproc}$) is the probability of generating an ADV_SCAN_IND within a *signaling processing period* or, as is the case in this situation, the probability that the scanner is in a *signaling processing period* (see Equation (6)).

(5) $$\bar{\tau_{sigproc}}=\left[\left(T_{IFS}+T_{scanREQ}+T_{IFS}\right)+\left(1-P_{NDScanREQ}^{col}\right)\cdot T_{scanRSP}+P_{NDScanREQ}^{col}\cdot T_{sync}\right]$$

(6) $$P_{NDAdvIND}^{sigproc}=\frac{\bar{T_{sigproc}}}{T_{advInterval}+\bar{\tau_{advDelay}}}\;\mathrm{with}\;\bar{T_{sigproc}}=\bar{N_{\det AdvIND}}\cdot \bar{\tau_{sigproc}}$$

*b. Decoding gaps.* These scanning interruptions appear when the scanning device processes a detected ADV_SCAN_IND, a detected SCAN_RSP, or is unable to detect an expected SCAN_RSP PDU after a specific timeout. *Decoding gaps* are added to the *signaling process gaps*. In a similar way to *signaling processing gaps*, the mean time that the scanner is involved in *decoding gaps* ($\bar{T_{decodGap}}$) also depends on the mean number of neighbor devices that complete the signaling process within an interval $T_{advInterval}+\bar{\tau_{advDelay}}$. The mean time is the result of the sum of several gaps linked to different events: post-processing of a correct or erroneous SCAN_RSP transmission ((a) in Equation (7)), post-processing of a *decoding gap* (blind time) after the timeout for the reception of the SCAN_RSP expires ((b) in Equation (7)), and post-processing of a *decoding gap* of an erroneous ADV_SCAN_IND transmission ((c) in Equation (7)). The characterization of the real chipset shows that an erroneous reception of the packet header, whose preamble has been detected, anticipates the trigger of a *decoding gap*. Thus, the analytical model considers both the gaps after the erroneous reception of the header part with probability $T_{sync}/T_{PDU}$, and the gaps after the complete reception of the PDU with probability $(T_{PDU}-T_{sync})/T_{PDU}$ (being $T_{PDU}$ equal to $T_{advIND}$ or $T_{scanRSP}$).

(7a) $$\bar{N_{detAdvIND}}\cdot \left(1-P_{NDScanREQ}^{col}\right)\cdot \left[\left(1-P_{NDScanRSP}^{col}\right)\cdot \bar{\tau_{decodGap}}+P_{NDScanRSP}^{col}\cdot \bar{\tau_{errDecodGap}{}^{*}}\right]\;with\;\;\bar{\tau_{errDecodGap}{}^{*}}=\frac{T_{scanRSP}-T_{sync}}{T_{scanRSP}}\cdot \bar{\tau_{errDecodGap}}+\frac{T_{sync}}{T_{scanRSP}}\cdot \max\left(0,\left(\bar{\tau_{errDecodGap}}-\left(T_{scanRSP}-T_{sync}\right)\right)\right)$$

(7b) $$+\bar{N_{detAdvIND}}\cdot P_{NDScanREQ}^{col}\cdot \bar{\tau_{errDecodGap}}$$

(7c) $$+\frac{\left(N_{BLE}-1-\bar{N_{detAdvIND}}\right)}{2}\cdot \frac{P_{NDAdvIND}^{col}\cdot (1-P_{NDAdvIND}^{gap})}{P_{NDAdvIND}^{}}\cdot \bar{\tau_{errDecodGap}{}^{**}}\;with\;\;\bar{\tau_{errDecodGap}{}^{**}}=\frac{T_{advIND}-T_{sync}}{T_{advIND}}\cdot \bar{\tau_{errDecodGap}}+\frac{T_{sync}}{T_{advIND}}\cdot \max\left(0,\left(\bar{\tau_{errDecodGap}}-\left(T_{advIND}-T_{sync}\right)\right)\right)\;Being$$

(7d) $$\bar{T_{decodGap}}=Eq.7(a)+Eq.7(b)+Eq.7(c)$$

Concerning the average time that the scanner is involved in a *decoding gap* after the erroneous reception of an ADV_SCAN_IND, this is obtained by multiplying the average time of these *decoding gaps* ($\bar{\tau_{errDecodGap}{}^{*}}$) by the average number of BLE devices for whose signals synchronization has been attempted, but which have not been detected due to a collision. That is, non-detected BLE devices due to gaps (signaling processing, decoding or periodic) are not considered. On the other hand, when a collision occurs, the scanner only tries to detect the preamble of the first arrived PDU. This means that only one colliding ADV_SCAN_IND will potentially generate a *decoding gap*. In a simplified approach, if we assume that a collision involves two advertising devices, the mean number of neighbor advertisers that are able to generate a *decoding gap* will be obtained by Equation (8). Note that $\left(N_{BLE}-1-\bar{N_{detAdvIND}}\right)$ is the number of neighbor advertisers whose ADV_SCAN_IND have not been detected; $\left[P_{NDAdvIND}^{col}\cdot (1-P_{NDAdvIND}^{gap})\right]/P_{NDAdvIND}^{}$, is the fraction of non-detections due exclusively to collisions ($P_{NDAdvIND}^{gap}$, which will be introduced next). Finally, the product of the two terms is divided by 2, because only one of the two advertisers involved in a collision generates a *decoding gap*.

(8) $$\frac{\left(N_{BLE}-1-\bar{N_{DetAdvIND}}\right)}{2}\cdot \frac{P_{NDAdvIND}^{col}\cdot (1-P_{NDAdvIND}^{gap})}{P_{NDAdvIND}^{}}$$

The ADV_SCAN_IND non-detection probability due to the *decoding gaps* ($P_{NDAdvIND}^{decodGap}$) is the probability of generating an ADV_SCAN_IND within a *decoding gap* period. This probability is equal to the probability that the scanner is in a *decoding gap* period, as is shown in Equation (9).

(9) $$P_{NDAdvIND}^{decodGap}=\frac{\bar{T_{decodGap}}}{T_{advInterval}+\bar{\tau_{advDelay}}}$$

*c. Periodic scanning gaps.* Assuming that there is only one scanning device, the probability of this type of gap ($P_{scanGap}^{pattern}$) is the quotient between the addition of the average durations of every gap occurring in the scan window (denoted as $\bar{T_{scanGap}}$) and $T_{scanWindow}$. $P_{scanGap}^{pattern}$ is obtained by Equation (10), given a number of gaps $N_{interFqChgGap}^{scanWindow}$ in a $T_{scanWindow}$ and derived by using $T_{scanWindow}$, $T_{fqChgGap}$_,_ $T_{interFqChgGap}$, $T_{gapInt1}$ and $T_{gapInt2}$ parameters. This characterization is generic, and applies for both types 1 and 2 real devices.

(10) $$P_{scanGap}^{pattern}=\frac{\bar{T_{scanGap}}}{\bar{T_{scanWindow}}}=\frac{\bar{T_{fqChgGap}}+N_{interFqChgGap}^{scanWindow}\cdot \bar{T_{interFqChgGap}}}{\bar{T_{scanWindow}}}$$

Once $P_{scanGap}^{pattern}$ is derived, we need to clarify how periodic gaps affect the PDU’s detection. We have seen that, once ADV_SCAN_IND reception is initiated, if the scanner has scheduled a periodic gap, two options can happen, with different results.

- Once a periodic gap is initiated, any transmission of ADV_SCAN_IND PDU that starts after the beginning of the periodic gap cannot be detected. Then, the time intervals between successive gaps are not modified.
- If ADV_SCAN_IND PDU packet reception begins before the start time of a planned periodic gap, there are significant differences between chipsets from different manufacturers. When type 1 scanner devices are evaluated, we measured that if an advertising event starts and the scanner has scheduled a periodic gap during the ADV_SCAN_IND reception, this gap is postponed at least until the reception is finished (no matter if the reception is correct or erroneous) or the *decoding gap* is initiated (if the PDU header is erroneous). Additionally, if the ADV_SCAN_IND reception is correct, or if the *periodic scanning gap* is planned to start after the ADV_SCAN_IND has been correctly received, the periodic gap is delayed until the end of the SCAN_RSP reception, or until the timeout on the SCAN_RSP reception is reached. It is clear that, in this situation, the interval from the delayed periodic gap and the following gap is shorter than the expected ones, in accordance with the pattern timing. Nevertheless, the following inter-gap intervals remain unchanged.

Furthermore, it is known that, after erroneous ADV_SCAN_IND receptions, or after successful SCAN_RSP receptions, the scanner introduces *decoding gaps.* In this case, the decoding gaps and also the delayed *scanning gap* should be planned to start simultaneously. Nevertheless, the largest of them is applied by the scanner. Furthermore, $P_{scanGap}^{pattern}$ remains unchanged, in accordance with Equation (10). Therefore, the ADV_SCAN_IND non-detection probability due to *periodic scanning gaps* ($P_{NDAdvIND}^{scanGap}$), in accordance with Equation (11), is the probability of transmitting an ADV_SCAN_IND in the *scanning gap* interval, which is equal to Equation (9).

(11) $$P_{NDAdvIND}^{scanGap}=P_{scanGap}^{pattern}=\frac{\bar{T_{fqChgGap}}+N_{interFqChgGap}^{scanWindow}\cdot \bar{T_{interFqChgGap}}}{\bar{T_{scanWindow}}}$$

Once $P_{NDAdvIND}^{scanGap}$ and $P_{NDAdvIND}^{decodGap}$ are calculated, the probability that the scanner is in a *scanning gap* has to be derived, regardless of whether the scanning gap was a *decoding gap* or a *periodic gap*. As these two effects are considered to be independent, we use the Equation (12) to compute the non-detection probability of ADV_SCAN_IND ($P_{NDAdvIND}^{gap}$) due to both effects.

(12) $$P_{NDAdvIND}^{gap}=P_{NDAdvIND}^{scanGap}+P_{NDAdvIND}^{decodGap}-P_{NDAdvIND}^{scanGap}\cdot P_{NDAdvIND}^{decodGap}$$

Then, the ADV_SCAN_IND non-detection probability due to the *scanning gaps* must be added to that due to the signaling processing period (see Equation (13)).

(13) $$P_{NDAdvIND}^{sigproc+gap}=P_{NDAdvIND}^{sigproc}+P_{NDAdvIND}^{gap}$$

Finally, the ADV_SCAN_IND non-detection probability due to collisions and all the effects explained above can be obtained by Equation (14).

(14) $$P_{NDAdvIND}^{}=P_{NDAdvIND}^{col}+\left(1-P_{NDAdvIND}^{col}\right)\cdot P_{NDAdvIND}^{sigproc+gap}$$

It is important to keep in mind that $P_{NDAdvIND}^{sigproc}$ and $P_{NDAdvIND}^{decodGap}$ components of $P_{NDAdvIND}^{sigproc+gap}$ depend on $P_{NDAdvIND}^{}$; and at the same time, $P_{NDAdvIND}^{sigproc}$, in addition to $P_{NDAdvIND}^{decodGap}$, will modify the probability $P_{NDAdvIND}^{}$. Therefore, an iterative resolution process according to Algorithm 1, will be used to obtain them.

Once $P_{NDAdvIND}^{}$ is obtained, $P_{NDScanREQ}^{}$ and $P_{NDScanRSP}^{}$ can be easily derived from Equations (15) and (16). Note that they are not affected by gaps.

(15) $$P_{NDScanREQ}^{}=1-(1-P_{NDAdvIND}^{})\cdot (1-P_{NDScanREQ}^{col})$$

(16) $$P_{NDScanRSP}^{}=1-(1-P_{NDAdvIND}^{})\cdot (1-P_{NDScanREQ}^{col})\cdot (1-P_{NDScanRSP}^{col})$$

Finally, the characterization of the propagation channel effects and the interference can be included easily in all the analysis through a Block Error Rate (BLER) parameter. In this case, all the equations used in the analytical characterization are the same, but using $P_{NDAdvIND}^{+BLER}$, $P_{NDScanREQ}^{col+BLER}$, $P_{NDScanRSP}^{col+BLER}$ (defined according to Equations (17)–(19), instead of $P_{NDAdvIND}^{}$, $P_{NDScanREQ}^{col}$, $P_{NDScanRSP}^{col}$).

(17) $$P_{NDAdvIND}^{+BLER}=P_{NDAdvIND}^{}+(1-P_{NDAdvIND}^{})\cdot BLER$$

(18) $$P_{NDScanREQ}^{col+BLER}=P_{NDScanREQ}^{col}+(1-P_{NDScanREQ}^{col})\cdot BLER$$

(19) $$P_{NDScanRSP}^{col+BLER}=P_{NDScanRSP}^{col}+(1-P_{NDScanRSP}^{col})\cdot BLER$$

**Algorithm 1. Iterative Resolution for Ideal and Type 1 Devices**
$\begin{array}{l} 1.\;n\leftarrow 0\;\mathrm{and}\;N_{ngdev}\leftarrow N_{BLE}-1\\ 2.\;\mathrm{obtain}\;P_{NDAdvIND}^{col},\;P_{NDScanREQ}^{col},\;P_{NDScanRSP}^{col}\;\mathrm{and}\;P_{NDAdvIND}^{scanGap}\;\mathrm{with}\;\mathrm{Equations}\;(1),\;(2),\;(3)\;\mathrm{and}\;(11)\\ 3.\;\mathrm{set}\;P_{NDAdvIND}^{}(0)\;\leftarrow P_{NDAdvIND}^{col}\;\\ 4.\;\mathrm{set}\;P_{NDAdvIND}^{gap}(0)\leftarrow 0\\ 5.\;n\leftarrow n+1;\\ 6.\;\mathrm{obtain}\;P_{NDAdvIND}^{sigproc}(n)\;\mathrm{with}\;\mathrm{Equations}\;(6)\;\mathrm{using}\;(5)\;\mathrm{and}\;(4)\;\mathrm{with}\;P_{NDAdvIND}^{}(n-1)\\ 7.\;\mathrm{obtain}\;P_{NDAdvIND}^{decodGap}(n)\;\mathrm{with}\;\mathrm{Equations}\;(9)\;\mathrm{using}\;(7)\;\mathrm{with}\;P_{NDAdvIND}^{}(n-1)\;\mathrm{and}\;P_{NDAdvIND}^{gap}(n-1)\\ 8.\;\mathrm{obtain}\;P_{NDAdvIND}^{sigproc+gap}(n)\;\mathrm{with}\;\mathrm{Equations}\;(13)\;\mathrm{and}\;(12)\;\mathrm{using}\;P_{NDAdvIND}^{sigproc}(n),\;P_{NDAdvIND}^{decodGap}(n)\;\mathrm{and}\;P_{NDAdvIND}^{scanGap}\\ 9.\;\mathrm{update}\;P_{NDAdvIND}^{}(n)\leftarrow P_{NDAdvIND}^{col}+\left(1-P_{NDAdvIND}^{col}\right)\cdot P_{NDAdvIND}^{sigproc+gap}(n)\\ 10.\;While\left(\;\left(\;P_{NDAdvIND}^{}(n)-P_{NDAdvIND}^{}(n-1)\right)>\varepsilon \right)\\ 11.\;\{\\ 12.\;n\leftarrow n+1;\\ 13.\;\mathrm{update}\;P_{NDAdvIND}^{sigproc}(n)\;\mathrm{with}\;\mathrm{Equations}\;(6)\;\mathrm{using}\;(5)\;\mathrm{and}\;(4)\;\mathrm{with}\;P_{NDAdvIND}^{}(n-1)\\ 14.\;\mathrm{update}\;P_{NDAdvIND}^{decodGap}(n)\;\mathrm{with}\;\mathrm{Equations}\;(9)\;\mathrm{using}\;(7)\;\mathrm{with}\;P_{NDAdvIND}^{}(n-1)\;\mathrm{and}\;P_{NDAdvIND}^{gap}(n-1)\\ 15.\;\mathrm{update}\;P_{NDAdvIND}^{sigproc+gap}(n)\;\mathrm{with}\;\mathrm{Equations}\;(13)\;\mathrm{and}\;(12)\;\mathrm{using}\;P_{NDAdvIND}^{sigproc}(n),\;P_{NDAdvIND}^{decodGap}(n)\;\mathrm{and}\;P_{NDAdvIND}^{scanGap}\\ 16.\;\mathrm{update}\;P_{NDAdvIND}^{}(n)\leftarrow P_{NDAdvIND}^{col}+\left(1-P_{NDAdvIND}^{col}\right)\cdot P_{NDAdvIND}^{sigproc+gap}(n)\\ 17.\;\mathrm{set}\;P_{NDAdvIND}(n)\leftarrow \;\left(P_{NDAdvIND}(n)+P_{NDAdvIND}(n-1)\right)/2\;\mathrm{to}\;\mathrm{assure}\;\mathrm{the}\;\mathrm{convergence}\;\\ 18.\;\}\\ 19.\;P_{NDAdvIND}\leftarrow P_{NDAdvIND}(n) \end{array}$


### 4.2. Non-Detection Probabilities for the Type 2 Chipsets

In this section, we describe the complete characterization of the non-detection probabilities for type 2 scanning devices. In fact, the model only requires the introduction of slight variations in the analysis described for both ideal and type 1 devices. So, we use the previous model, while emphasizing the required adaptations. There are two main differences between types 1 and 2 devices. 

The first one concerns the *decoding gaps*. In this case, the processing *decoding gap* after the timeout for the reception of the SCAN_RSP expires is not required. The scanner enters scan mode. That is, the mean time that the scanner is involved in *decoding gaps* ($\bar{T_{decodGap}}$), used to compute the $P_{NDAdvIND}^{decodGap}$ (Equation (20)) is equivalent to Equation (9), with the (a) and (c) components described in Equation (7).

(20) $$\bar{T_{decodGap}}=Eq.7(a)+Eq.7(c)$$

The second difference is the impact of *periodic scanning gaps*. Contrary to type 1 scanning devices, if the ADV_SCAN_IND reception prior to the beginning of a periodic gap, it is necessary to include the following cases:

The same as with type 1 scanning devices, if the scanner has planned to start a periodic gap during the ADV_SCAN_IND reception time, the pause is delayed at least up until the reception is finished (no matter whether the reception is correct or erroneous) or the *decoding gap* is initiated (when the header part of the PDU is erroneous). However, if the scanner has planned to start a periodic gap during the subsequent $T_{IFS}$, the pause is delayed until the $T_{IFS}$ is finished and the discovery event is closed in that frequency. The main difference occurs if the scanner has planned a periodic gap in the time interval between the end of the first $T_{IFS}$ interval (between the ADV_SCAN_IND and the SCAN_REQ transmission) and the time the scanner expects to finish the reception of the SCAN_RSP. In this case, the scanner interrupts its operation until the time the periodic gap is planned, and then executes the periodic gap. That is, the discovery event is closed. Note that, from the point of view of the scanner, the expected duration of the SCAN_RSP will be the maximum allowed ($T_{scanRSP}^{Max}$). 

Assuming all that, we can conclude that any periodic gap planned along the maximum time involving the discovery process (that is, $T_{advIND}+T_{sigproc}^{Max}$, being $T_{sigproc}^{Max}=T_{IFS}+T_{scanREQ}+T_{IFS}+T_{scanRSP}^{Max}$) modifies the mean duration of the *signaling processing period (*$\bar{T_{sigproc}}$).

In order to obtain $\bar{T_{sigproc}}$, we firstly obtain the probability of having a periodic gap within the $T_{advIND}+T_{sigproc}^{Max}$ (defined as $P_{patternScanGap}^{MaxSigproc}$ in Equation (22)) by multiplying the rate of periodic gaps ($R_{scanGap}^{pattern}$), obtained by Equation (21), by $T_{advIND}+T_{sigproc}^{Max}$.

(21) $$R_{scanGap}^{pattern}=\frac{1+N_{interFqChgGap}^{scanWindow}}{\bar{T_{scanWindow}}}$$

(22) $$P_{patternScanGap}^{MaxSigproc}=R_{scanGap}^{pattern}\cdot \left(T_{AdvIND}+T_{sigproc}^{Max}\right)$$

$\bar{T_{sigproc}}$ is obtained by Equation (23), using $\bar{\tau_{sigproc}}$, as defined in Equation (5). Then, we derive $P_{NDAdvIND}^{sigproc}$ with Equation (24).

(23) $$\bar{T_{sigproc}}=\bar{N_{detAdvIND}}\cdot \left[(1-P_{patternScanGap}^{MaxSigproc})\cdot \bar{\tau_{sigproc}}+P_{patternScanGap}^{MaxSigproc}\cdot \left(T_{IFS}+\frac{\left(T_{scanREQ}+T_{IFS}+T_{scanRSP}^{Max}\right)}{2}\right)\right]$$

(24) $$P_{NDAdvIND}^{sigproc}=\frac{\bar{T_{sigproc}}}{T_{advInterval}+\bar{\tau_{advDelay}}}$$

Beyond the differences in the calculation of $P_{NDAdvIND}^{sigproc}$ and $\;P_{NDAdvIND}^{decodGap}$, the process of computing $P_{NDAdvIND}^{}$ is analogous to that used for ideal and type 1 scanning devices. In this case, the iterative resolution process is rewritten in Algorithm 2.

Once $P_{NDAdvIND}^{}$ is obtained, $P_{NDScanREQ}^{}$ and $P_{NDScanRSP}^{}$ can be easily derived from Equations (25) and (26).

(25) $$P_{NDScanREQ}^{}=1-(1-P_{NDAdvIND}^{})\cdot (1-P_{NDScanREQ}^{col})\cdot (1-P_{patternScanGap}^{MaxSigproc})$$

(26) $$P_{NDScanRSP}^{}=1-(1-P_{NDAdvIND}^{})\cdot (1-P_{NDScanREQ}^{col})\cdot (1-P_{NDScanRSP}^{col})\cdot (1-P_{patternScanGap}^{MaxSigproc})$$

Finally, the effects of interference and channel response can be easily introduced in all the analysis through a BLER parameter, in a similar way to that described in Section 4.1.

**Algorithm 2. Iterative Resolution for Type 2 Devices**
$\begin{array}{l} 1.\;n\leftarrow 0\;\mathrm{and}\;N_{ngdev}\leftarrow N_{BLE}-1\\ 2.\;\mathrm{obtain}\;P_{NDAdvIND}^{col},\;P_{NDScanREQ}^{col},\;P_{NDScanRSP}^{col},\;P_{NDAdvIND}^{scanGap}\;\mathrm{and}\;P_{patternScanGap}^{MaxSigproc}\mathrm{with}\;\mathrm{Equations}\;(1),\;(2),\;(3),\;(11)\;\mathrm{and}\;(22)\\ 3.\;\mathrm{set}\;P_{NDAdvIND}^{}(0)\;\leftarrow P_{NDAdvIND}^{col}\;\\ 4.\;\mathrm{set}\;P_{NDAdvIND}^{gap}(0)\leftarrow 0\\ 5.\;n\leftarrow n+1;\\ 6.\;\mathrm{obtain}\;P_{NDAdvIND}^{sigproc}(n)\;\mathrm{with}\;\mathrm{Equations}\;(24)\;\mathrm{using}\;(23),\;(5)\;\mathrm{and}\;(4)\;\mathrm{with}\;P_{NDAdvIND}^{}(n-1)\\ 7.\;\mathrm{obtain}\;P_{NDAdvIND}^{decodGap}(n)\;\mathrm{with}\;\mathrm{Equations}\;(9)\;\mathrm{using}\;(20)\;\mathrm{with}\;P_{NDAdvIND}^{}(n-1)\;\mathrm{and}\;P_{NDAdvIND}^{gap}(n-1)\\ 8.\;\mathrm{obtain}\;P_{NDAdvIND}^{sigproc+gap}(n)\;\mathrm{with}\;\mathrm{Equations}\;(13)\;\mathrm{and}\;(12)\;\mathrm{using}\;P_{NDAdvIND}^{sigproc}(n),\;P_{NDAdvIND}^{decodGap}(n)\;\mathrm{and}\;P_{NDAdvIND}^{scanGap}\\ 9.\;\mathrm{update}\;P_{NDAdvIND}^{}(n)\leftarrow P_{NDAdvIND}^{col}+\left(1-P_{NDAdvIND}^{col}\right)\cdot P_{NDAdvIND}^{sigproc+gap}(n)\\ 10.\;While\left(\;\left(\;P_{NDAdvIND}^{}(n)-P_{NDAdvIND}^{}(n-1)\right)>\varepsilon \right)\\ 11.\;\{\\ 12.\;n\leftarrow n+1;\\ 13.\;\mathrm{update}\;P_{NDAdvIND}^{sigproc}(n)\;\mathrm{with}\;\mathrm{Equations}\;(24)\;\mathrm{using}\;(23),\;(5)\;\mathrm{and}\;(4)\;\mathrm{with}\;P_{NDAdvIND}^{}(n-1)\\ 14.\;\mathrm{update}\;P_{NDAdvIND}^{decodGap}(n)\;\mathrm{with}\;\mathrm{Equations}\;(9)\;\mathrm{using}\;(20)\;\mathrm{with}\;P_{NDAdvIND}^{}(n-1)\;\mathrm{and}\;P_{NDAdvIND}^{gap}(n-1)\\ 15.\;\mathrm{update}\;P_{NDAdvIND}^{sigproc+gap}(n)\;\mathrm{with}\;\mathrm{Equations}\;(13)\;\mathrm{and}\;(12)\;\mathrm{using}\;P_{NDAdvIND}^{sigproc}(n),\;P_{NDAdvIND}^{decodGap}(n)\;\mathrm{and}\;P_{NDAdvIND}^{scanGap}\\ 16.\;\mathrm{update}\;P_{NDAdvIND}^{}(n)\leftarrow P_{NDAdvIND}^{col}+\left(1-P_{NDAdvIND}^{col}\right)\cdot P_{NDAdvIND}^{sigproc+gap}(n)\\ 17.\;\mathrm{set}\;P_{NDAdvIND}(n)\leftarrow \;\left(P_{NDAdvIND}(n)+P_{NDAdvIND}(n-1)\right)/2\;\mathrm{to}\;\mathrm{assure}\;\mathrm{the}\;\mathrm{convergence}\\ 18.\;\}\\ 19.\;P_{NDAdvIND}\leftarrow P_{NDAdvIND}(n) \end{array}$


### 4.3. Derived Parameters of Interest

To compare both the standard and interrupted versions of the scannable undirected advertising event and non-connectable advertising event, with only advertising PDUs (named in the specifications as ADV_NONCONN_IND and previously studied in [13]), the main parameters of interest are the average time required to discover all devices based on SCAN_REQ detection ($\bar{D_{allDet}^{scanREQ}}$) and the average time required to discover all devices based on ADV_NONCONN_IND or in ADV_SCAN_IND ($\bar{D_{allDet}^{advIND}}$). The aim is that $\bar{D_{allDet}^{scanREQ}}$ applies in scannable undirected advertising events, whereas $\bar{D_{allDet}^{advIND}}$ applies in non-connectable advertising events, with only advertising PDUs. Nevertheless, the comparison is not fair, because when only ADV_NONCONN_IND are sent, the advertisers do not really know if they have been discovered. In order to perform a fairer comparison, assuming that we are really interested in knowing when the advertiser is aware of being discovered by the scanner, we can also obtain $\bar{D_{allDet}^{advIND}}$ for scannable undirected advertising events. In this case, this parameter is calculated when the ADV_SCAN_IND is detected.

First, we can easily develop an approach for deriving the bound for $\bar{D_{allDet}^{scanREQ}}$, when advertisers stop the discovery process once they have successfully received a SCAN_REQ. Detection delay for the *nth* detected device depends on the $P_{NDScanREQ}^{}$ probability, which changes over time, as neighbor devices are discovered and stop their advertising events. As an exact characterization makes the analysis too complex for practical utility, we only derive a simple but accurate bound. The basis of the analysis, described in Algorithm 3, is simple. For each time interval between advertisements of a reference advertiser ($T_{advInterval}+T_{advDelayMax}$), we assume that the number of undiscovered devices in the system remains fixed. Starting from the number of devices present in the scenario ($N_{BLE}$), we initialize the number of detected devices ($N_{devDet}$) to zero. Then, sequentially, we compute $P_{NDScanREQ}^{}$ according to the analytical models previously described. Once $P_{NDScanREQ}^{}$ is obtained, we can derive the mean number of devices whose SCAN_REQ can be detected ($\bar{N_{detScanREQ}}$) in each time interval. Note that the non-detection probabilities obtained in Section 4.1 and 4.2 implied a characterization of the mean number of detected devices in each advertising interval. Thus, the approach is adequate. Each round, we increase the number of detected devices ($N_{devDet}$) and decrease the number of neighbor devices ($N_{ngdev}$) in the computed $\bar{N_{detScanREQ}}$ quantity, while the delay is increased by $T_{advInterval}+T_{advDelayMax}$. This process is repeated until only one device remains active. The last device is expected to generate a new advertisement with a mean delay of $\left(T_{advInterval}+T_{advDelayMax}\right)/2$. On the other hand, even if only one advertiser is present, the mean time between the transmission of an advertising packet until the scanner correctly receives it, is $\bar{t_{advEvent}}\cdot P_{NDScanREQ}^{}/\left(1-P_{NDScanREQ}^{}\right)$.

**Algorithm 3. Algorithm for Deriving the Bound for**
$\bar{D_{allDet}^{scanREQ}}$
**, When Advertisers Stop the Discovery Process Once They Have Successfully Received a SCAN_REQ**
$\begin{array}{l} 1.\;\mathrm{set}\;N_{devDet}\leftarrow 0,\;D_{\det}\leftarrow 0\;\mathrm{and}\;N_{ngdev}\leftarrow N_{BLE}-1-N_{devDet}\\ 2.\;While\left(\;N_{devDet}<N_{BLE}-1\right)\\ 3.\;\{\\ 4.\;\mathrm{obtain}\;P_{NDAdvIND}\mathrm{with}\;\mathrm{Algorithm}\;1\;\mathrm{or}\;\mathrm{Algorithm}\;2\;\mathrm{and}\;N_{ngdev}\\ 5.\;P_{NDScanREQ}^{}=1-(1-P_{NDAdvIND}^{})\cdot (1-P_{NDScanREQ}^{col})\;\\ 6.\;\bar{N_{detScanREQ}}=\sum_{i=1}^{N_{ngdev}+1}i\cdot \left(\begin{array}{l} N_{ngdev}+1\\ i \end{array}\right){\left(1-P_{NDScanREQ}^{}\right)}^{i}\cdot {\left(P_{NDScanREQ}^{}\right)}^{\left(N_{ngdev}+1\right)-i}\\ 7.\;N_{devDet}\leftarrow N_{devDet}+\left\lfloor \bar{N_{detScanREQ}}\right\rfloor \\ 8.\;D_{\det}\leftarrow D_{\det}+\left(T_{advInterval}+T_{advDelayMax}\right)\\ 9.\;N_{ngdev}\leftarrow N_{BLE}-1-N_{devDet}\\ 10.\;\}\\ 11.\;\mathrm{obtain}\;P_{NDScanREQ}^{}\;\mathrm{when}\;N_{BLE}=1\\ 12.\;\bar{D_{allDet}^{scanREQ}}\leftarrow D_{\det}+\frac{\left(T_{advInterval}+T_{advDelayMax}\right)}{2}+\frac{P_{NDScanREQ}^{}}{1-P_{NDScanREQ}^{}}\cdot \bar{t_{advEvent}}+(T_{advIND}+T_{IFS}+T_{scanREQ}) \end{array}$


Alternatively, and for comparison purposes, we can derive a bound for $\bar{D_{allDet}^{scanREQ}}$ when the advertisers do not stop the advertising process after they have been discovered. In this case (see Algorithm 4), each $T_{advInterval}+T_{advDelayMax}$ interval, the number of devices that may be detected is always the same. Thus, we only need to obtain the number of devices among the detected ones that have not been previously discovered. The process can be applied to derive $\bar{D_{allDet}^{advIND}}$ by only considering $P_{NDAdvIND}^{}$ and $\bar{N_{detAdvIND}}$, instead of $P_{NDScanREQ}^{}$ and $\bar{N_{detScanREQ}}$.

**Algorithm 4. Algorithm for Deriving a Bound for $\bar{D_{allDet}^{scanREQ}}$ When the Advertisers Do Not Stop the Advertising Process After They Have Been Discovered**
$\begin{array}{l} 1.\;\mathrm{set}\;N_{devDet}\leftarrow 0,,\;D_{\det}\leftarrow 0\;\mathrm{and}\;N_{ngdev}\leftarrow N_{BLE}-1\\ 2.\;\mathrm{obtain}\;P_{NDAdvIND}\mathrm{with}\;\mathrm{Algorithm}\;1\;\mathrm{or}\;\mathrm{Algorithm}\;2\;\mathrm{and}\;N_{ngdev}\\ 3.\;P_{NDScanREQ}^{}=1-(1-P_{NDAdvIND}^{})\cdot (1-P_{NDScanREQ}^{col})\\ 4.\;\bar{N_{detScanREQ}}=\sum_{i=1}^{N_{ngdev}+1}i\cdot \left(\begin{array}{l} N_{ngdev}+1\\ i \end{array}\right){\left(1-P_{NDScanREQ}^{}\right)}^{i}\cdot {\left(P_{NDScanREQ}^{}\right)}^{\left(N_{ngdev}+1\right)-i}\\ 5.\;N_{devDet}\leftarrow N_{devDet}+\left\lfloor \bar{N_{detScanREQ}}\right\rfloor \\ 6.\;D_{\det}\leftarrow D_{\det}+\left(T_{advInterval}+T_{advDelayMax}\right)\\ 7.\;While\;\left(N_{devDet}<N_{BLE}-1\right)\\ 8.\;\{\;\\ 9.\;P_{newDevDet}=1-N_{devDet}/N_{BLE};\\ 10.\;\bar{N_{newDevDet}}=\sum_{i=1}^{\left\lfloor \bar{N_{detScanREQ}}\right\rfloor }i\cdot \left(\begin{array}{l} \left\lfloor \bar{N_{detScanREQ}}\right\rfloor \\ i \end{array}\right){\left(P_{newDevDet}\right)}^{i}\cdot {\left(1-P_{newDevDet}\right)}^{\left(\left\lfloor \bar{N_{detScanREQ}}\right\rfloor \right)-i}\\ 11.\;N_{devDet}\leftarrow N_{devDet}+\bar{N_{newDevDet}}\\ 12.\;D_{\det}\leftarrow D_{\det}+\left(T_{advInterval}+T_{advDelayMax}\right)\\ 13.\;\}\\ 14.\;\bar{D_{allDet}^{scanREQ}}\leftarrow D_{\det}+\frac{\left(T_{advInterval}+T_{advDelayMax}\right)}{2}+\frac{P_{NDScanREQ}^{}}{1-P_{NDScanREQ}^{}}\cdot \bar{t_{advEvent}}+(T_{advIND}+T_{IFS}+T_{scanREQ}) \end{array}$


We also have interest in deriving other parameters similar to those obtained for the non-connectable advertising events, with only ADV_NONCONN_IND, previously characterized in [13]. In order to introduce the parameters in a generalized form, they are referred to a generic xpdu, and must be replaced by *advIND, scanREQ* and *scanRSP*. 

The *average number of transmissions of a specific type of PDU* (i.e., ADV_SCAN_IND, SCAN_REQ or SCAN_RSP) *required before detection* ($\bar{N_{req}^{xpdu}}$) can be straightforwardly obtained by Equation (27).

(27) $$\bar{N_{req}^{xpdu}}=1\cdot \left(1-P_{NDxpdu}^{}\right)+\sum_{k=1}^{\infty }\left(1+k\right)\cdot (1-P_{NDxpdu}^{})\cdot P_{NDxpdu}^{}{}^{k}=1+\frac{P_{NDxpdu}^{}}{\left(1-P_{NDxpdu}^{}\right)}$$

We define the *average detection delay* ($\bar{D_{\det ect}^{xpdu}}$) as the mean time interval between the transmission of the first xpdu (i.e., ADV_SCAN_IND, SCAN_REQ or SCAN_RSP) packet by the advertiser, and the correct reception of these xpdus correctly using Equation (28).

(28) $$\bar{D_{detect}^{xpdu}}=(\bar{N_{req}^{xpdu}}-1)\cdot \bar{t_{advEvent}}$$

Using Equation (27), we can obtain the mean time *between two consecutive detections* with Equation (29).

(29) $$\bar{t_{interDetect}^{xpdu}}=\bar{N_{req}^{xpdu}}\cdot \bar{t_{advEvent}}$$

Finally, the mean number of advertiser detections within a window of coverage, $T_{covWindow}$, or alternatively, a time threshold for detection under the coverage area, $D_{TH}$ ($D_{TH}<T_{covWindow}$), is

(30) $$\bar{N_{detect}^{xpdu}}=\frac{\min(D_{TH},T_{covWindow}^{})}{\bar{t_{interDetect}^{xpdu}}}$$

## 5. Performance Evaluation

The device discovery process for BLE based on non-connectable advertising events with only advertising PDUs is fairly simple. This is the reason why we explored the use of this process to discover a high number of users in a short time period in [13]. This requirement concerns potential applications, such as the sport ones mentioned in Section 1. Scannable undirected advertising events were excluded in [13] due to the expected lower discovering capacities associated with higher signaling traffic and, thus, their higher collision probabilities. However, the possibility of stopping the discovery process after a successful detection of an SCAN_REQ makes this option more attractive in Bluetooth version 5.0. Thus, in accordance with these potential applications requirements, we want to compare and quantify the discovery capacities for the three possible configurations: discovering process with ADV_NONCONN_IND only, and continuous and interrupted processes with SCAN_REQ and SCAN_RSP. For evaluation purposes, the main parameters included in the analysis are:

- Non-detection probabilities of ADV_SCAN_IND (or alternatively ADV_NONCONN_IND), SCAN_REQ and SCAN_RSP, since they determine the overall non-detection probability and they are involved in the determination of the average time required to discover all the devices based on SCAN_REQ detection ($\bar{D_{allDet}^{scanREQ}}$), and the average time required to discover all the devices based on ADV_SCAN_IND detection ($\bar{D_{allDet}^{advIND}}$).
- The average time required to discover all the devices based on SCAN_REQ detection ($\bar{D_{allDet}^{scanREQ}}$) and the average time required to discover all the devices based on ADV_SCAN_IND detection ($\bar{D_{allDet}^{advIND}}$) or ADV_NONCONN_IND detection.
- Probability that all the devices are detected within a window of opportunity or a time threshold for detection under the coverage area, $D_{TH}$ ($D_{TH}<T_{covWindow}$).

The performance of the BLE discovery process, and particularly the tradeoff between discovery capabilities versus energy consumption of the scanner, greatly depends on the selected scanner parameter settings ($T_{scanInterval}$ and $T_{scanWindow}$ values), in addition to the advertising interval and the advertising PDU size. Once a scanning interval value is set in the scanner, its energy consumption decreases as long as the $T_{scanWindow}$ decreases, whereas the non-detection probability increases. Nevertheless, we have fixed $T_{scanInterval}=T_{scanWindow}$, because the goal is to detect the highest number of BLE devices in the shortest time interval. Firstly, the analysis, simulations and experiments are done in almost ideal conditions. Experiments are configured in controlled conditions, without the presence of interferences and low channel losses. After verifying that the simulation and the mathematical model meet the results obtained in the experimental tests, both the model and the simulation tool allow us to extend the analysis to a higher number of devices and several channel/interference conditions. Concerning the advertisers, in this section we analyze the impact of real peculiarities of the BLE chipsets on the discovery capacities, and the impact of $T_{advInterval}$ and ADV_NONCONN_IND/ADV_SCAN_IND PDUs sizes.

### Results

We developed a simulator in C++ that fully reproduces (without any simplification) both the advertising process in according with BLE specification and the real scanner configurations according to the peculiarities described Section 3. In order to obtain the performance statistics, we averaged up to 10,000 coverage time intervals. Errors due to interference or channel loss conditions can be considered. However, to reproduce the experimental conditions, results are obtained in ideal conditions (i.e., BLER = 0%). As mentioned above, the experimental testbed was configured in controlled conditions to make these effects negligible. Specifically, the advertising BLEs transmit with a power level of 4 dBm. In a scenario with only one advertising BLE, we verified that with 4 dBm and with a power transmission level of −40 dBm, the non-detection probability corresponds to $P_{NDAdvIND}^{scanGap}$ and $P_{NDScanRSP}^{scanGap}$, respectively. Thus, the BLER effects are almost negligible and the assumption of ideal conditions is suitable. In a real operating scenario, the time interval where an advertiser and the scanner are under mutual coverage may vary from one advertiser to another, as there may be times when the link is obstructed by obstacles (for example, a runner may be obstructed by other runners). It is important that the scanner is placed in a position with a good view of the entire area to be covered. In addition, our study considered the worst-case scenario, in which any overlap between two received packets results in the loss of both. In practice, there would be a capture effect, so that a good number of collisions would allow correct decoding of one of the packets. In any case, all this affects all discovery methods, not only the one proposed in this paper, and in no way is our proposal more affected by these situations. The most important parameters used in the evaluation are summarized in Table 3. 

**Figure 7:**
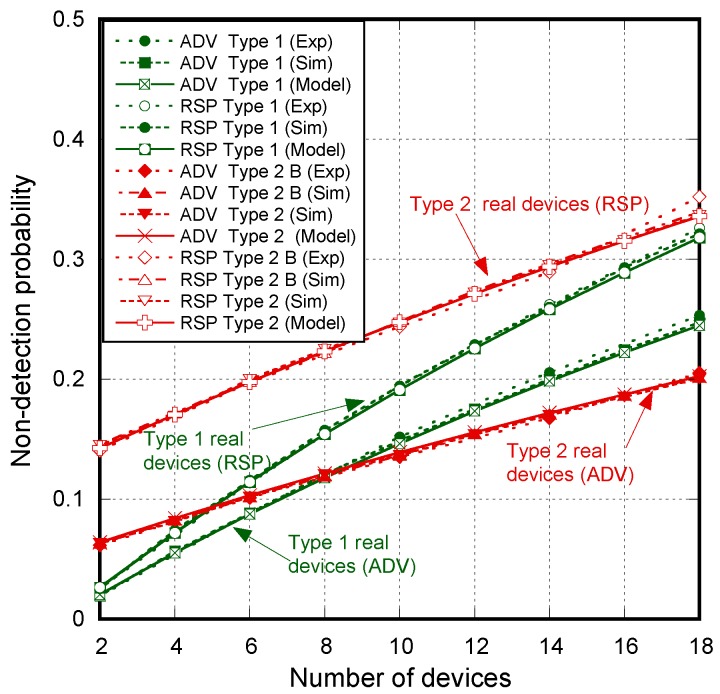
ADV_SCAN_IND (ADV) and SCAN_RSP (RSP) non-detection probabilities for types 1 and 2 scanning devices and scannable undirected advertising, with backoff (denoted by B) and without backoff implementation. Comparison between experimental measurements (Exp), simulation (Sim) and the analytical model (Model) for $T_{advIND}=T_{scanREQ}=$ 176 μs, $T_{scanRSP}=$ 152 μs with $T_{advInterval}=$ 100 ms and $T_{advDelayMax}=$ 10 ms.

Figure 7 shows the non-detection probabilities of ADV_SCAN_IND ($P_{NDAdvIND}^{}$) and SCAN_RSP ($P_{NDScanREQ}^{}$), when a continuous scannable undirected advertising event involving SCAN_REQ and SCAN_RSP is considered. The aim was to compare the simulation results (Sim) and the mathematical model results (Model), obtained when real scanning devices are assumed (types 1 and 2 scanning devices are denoted as types 1 and 2, respectively), with the experimental measurement results (Exp), as the number of advertisers increases from 2 to 18. Results are obtained for $T_{advIND}=T_{scanREQ}=$176 μs, $T_{scanRSP}=$ 152 μs with $T_{advInterval}=$ 100 ms and $T_{advDelayMax}=$10 ms. We can see that the experimental results are in perfect agreement with the mathematical model and the simulations. Thus, we can assume that the scanner is well characterized, and the mathematical model is accurate. Note that the analytical model and both the simulation and experimental results for type 1 scanning devices do not implement the backoff algorithm. Nevertheless, for type 2 scanning devices, Figure 7 includes experimental and simulation results taking into account the backoff algorithm (denoted by B in theFigure 7), in addition to the results obtained by simulation and by the analytical model without backoff implementation. We note that results are analogous because the backoff effect is almost negligible when the number of devices involved in the scenario is low.

**Figure 8:**
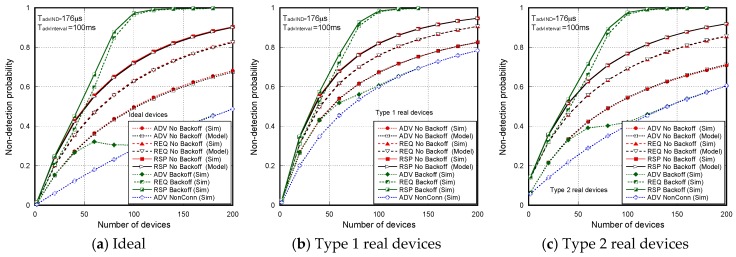
ADV_SCAN_IND (ADV), SCAN_REQ (REQ), SCAN_RSP (RSP) and ADV_NONCONN_IND (ADV NonConn) non-detection probabilities for scannable undirected and non-connectable advertising events, with and without backoff (denoted by Backoff and No Backoff) implementation, as the number of advertisers increases. Comparison between the mathematical model and the simulation for ideal (**a**), type 1 (**b**) and type 2 (**c**) scanning devices, for $T_{advIND}=T_{scanREQ}=$ 176 μs, $T_{scanRSP}=$ 152 μs with $T_{advInterval}=$ 100 ms and $T_{advDelayMax}=$ 10 ms.

By comparing the results of both ADV_SCAN_IND and SCAN_RSP non-detection probabilities, we can observe that differences between the actual implementations are quite significant and have to be considered. In this case, and considering the ADV_SCAN_IND non-detection probability, the type 1 scanner has a better performance than the type 2 up to eight advertisers, with the type 2 device being better for higher values. Nevertheless, the advantages of the type 2 real device in terms of ADV_SCAN_IND non-detection probability are not maintained when SCAN_RSP is considered. As we explained above, in type 2 scanning devices, if a periodic gap is planned between the reception of the ADV_SCAN_IND and the time the scanner expects to finish the reception of the SCAN_RSP, the scanner interrupts its operation until the time the periodic gap is planned, and then executes the periodic gap. This results in higher SCAN_REQ and SCAN_RSP non-detection probabilities, when the number of BLE advertisers is low. However, as shown in Figure 8, the weight of this effect decreases as the number of advertisers grows, and type 2 scanner is a better choice for these parameter settings when a large number of advertisers coexist.

Figure 8 extends the comparison performed in Figure 7 for a higher number of advertisers, $N_{BLE}$ up to 200. ADV_SCAN_IND, SCAN_REQ and SCAN_RSP non-detection probabilities results, obtained by simulation, for the scannable undirected advertising with backoff (denoted by ADV/RSP/REQ Backoff) and without backoff implementation (denoted as ADV/RSP/REQ No backoff), are compared with the ADV_NONCONN_IND non-detection probability for non-connectable advertising events (denoted as NonConn). The comparison is performed for ideal (a), type 1 (b) and type 2 (c), assuming the same configuration ($T_{advIND}=T_{scanREQ}=$ 176 μs, $T_{scanRSP}=$ 152 μs with $T_{advInterval}=$ 100 ms and $T_{advDelayMax}=$ 10 ms). The analytical model results for scannable undirected advertising without backoff are also included. Firstly, we notice that the analytical model nearly matches with the results of the simulations for the whole range of devices when no backoff implementation is considered. If we narrow the focus of the analysis to scannable undirected advertising, it is evident that the differences between actual devices and the ideal implementation cannot be ignored. As already mentioned, for a higher number of advertisers, if no backoff implementation is considered, type 2 scanning devices offer better results than type 1 in terms of SCAN_REQ non-detection probabilities, even though this advantage is not as significant as when ADV_SCAN_IND is compared. If backoff implementation is included, we realize the negative and highly limiting impact of this mechanism in all cases. Note that, as we explained in Section 3, backoff is only present in type 2 scanning devices, but it has been included in type 1 devices for comparison purposes. In this case, SCAN_REQ and SCAN_RSP non-detection probabilities grow, because SCAN_REQ are actually prevented from being transmitted (collisions between PDUs grow and thus *upperLimit* of the backoff algorithm is doubled until it reaches its maximum, 256, on many occasions). Accordingly, ADV_SCAN_IND non-detection probabilities for the scannable undirected advertising converge to values similar to those obtained with non-connectable advertising events, as the number of SCAN_REQ tends to zero in scenarios with a high number of devices. The backoff mechanism is not really required in a scenario with only one scanner, but the mandatory implementation unnecessarily degrades the discovering capacities for a larger number of devices, if the SCAN_REQ reception is considered as criterion. In fact, when backoff is used and the number of advertisers exceeds a value of about 100 (for the set of parameters defined), the discovering capacities of scannable undirected and non-connectable advertising event will be very similar, from a ADV_SCAN_IND or ADV_NONCONN_IND reception perspective. Nevertheless, the use of a scannable undirected advertising scheme makes no sense, given that it does not work as it should. Thus, potential enhancements could include better-adapted designs of the backoff process, or even deactivation under certain conditions. In the end, type 1 scanning devices, even though it seems that they do not meet the standard recommendations (i.e., do not implement backoff), offer the best results. 

In any case, and as expected, if we compare the schemes only from the advertising packet non-detection probability perspective, it is clear that the standard continuous scannable undirected advertising offers lower discovery capacities than the non-connectable option. So, we extend the analysis to the proposed adapted version of scannable undirected advertising.

**Figure 9:**
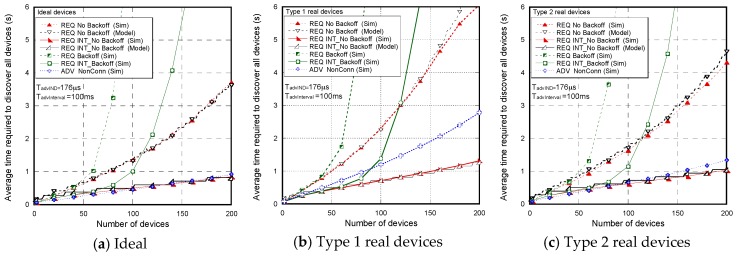
Average time required to discover all devices (in seconds), based on ADV_NONCONN_IND (ADV NonConn) for non-connectable advertising events and SCAN_REQ (REQ) reception for scannable undirected advertising events (standard and interrupted versions) with and without backoff, as the number of advertisers increases. Comparison between the mathematical model and the simulation for ideal (**a**), type 1 (**b**) and type 2 (**c**) scanning devices, for $T_{advIND}=T_{scanREQ}=$ 176 μs, $T_{scanRSP}=$ 152 μs with $T_{advInterval}=$ 100 ms and $T_{advDelayMax}=$ 10 ms.

In order to do that, Figure 9 extends the comparison performed in Figure 8 to the average time required to discover all the devices based on SCAN_REQ for the scannable undirected advertising event and ADV_NONCONN_IND reception for non-connectable advertising event. The comparison is not fair, because SCAN_REQ reception is a criterion more restrictive than ADV_SCAN_IND reception, but we consider that the reception of SCAN_REQ is a valuable indicator in the new scheme. Once a SCAN_REQ has been received by an advertiser, we can be assured that both the advertiser and the scanner realize that the device has been discovered. If only ADV_SCAN_IND reception is used, the device has been discovered, but the advertiser is not aware. Now, simulation and analytical model results are obtained for the continuous advertising event with SCAN_REQ and SCAN_RSP, whereas only the simulation is shown for the schemes when the advertiser interrupts the advertising process (denoted as INT) after correctly receiving an SCAN_REQ and when non-connectable advertising events with only ADV_NONCONN_IND are considered. We see that the upper bound (derived analytically) for the average time required to discover all the devices closely matches the simulation results, both for the interrupted and continuous version of the scannable undirected advertising events. The backoff implementation has a severe impact on SCAN_REQ reception. For example, results show that the interrupted version without backoff clearly offers better results than the non-connectable advertising scheme, previously analyzed in [13], when real devices are considered, particularly for type 1 devices. In the ideal implementation, differences are almost negligible from the mean delay point of view. Thus, it is clear that real chipset implementation needs to be considered in any evaluation. Until now, the adapted scannable indirect scheme not only reduces the time required but also the energy consumption of advertisers. On the other hand, advertisers are aware that they have been discovered. 

**Figure 10:**
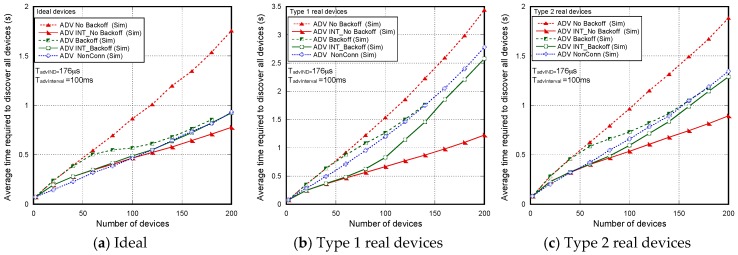
Average time required to discover all devices (in seconds), based on ADV_SCAN_IND (ADV) reception for scannable undirected (standard and interrupted versions) and non-connectable advertising events ADV_NONCONN_IND (ADV NonConn), with and without backoff, as the number of advertisers increases. Comparison between ideal (**a**), type 1 (**b**) and type 2 (**c**) scanning devices, for $T_{advIND}=T_{scanREQ}=$ 176 μs, $T_{scanRSP}=$ 152 μs with $T_{advInterval}=$ 100 ms and $T_{advDelayMax}=$ 10 ms.

The same analysis, based on a fairer discovery indicator (the ADV_SCAN_IND or ADV_NONCONN_IND) reception for both scannable undirected and non-connectable advertising events) is performed in Figure 10. We see that the interrupted version without backoff clearly offers the best results in all cases, with type 2 devices (without backoff) being the more attractive option. On the other hand, although we have excluded the backoff implementation, the interrupted version with backoff offers similar results to non-connectable advertising events for scenarios with a very high number of devices. Nevertheless, in order to compare the proposals, not only the average time required to discover all the devices, but also the distribution, is important. Therefore, we compute the probability that not all the devices are detected before a time threshold ($D_{TH}$). Note that discovery capacity depends on $D_{TH}$, so it should be evaluated for each desired application by considering the appropriate value for $D_{TH}$.

**Figure 11:**
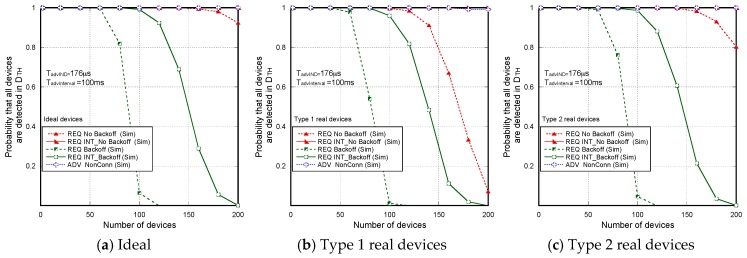
Probability that all devices are detected in $D_{TH}$ ($D_{TH}=5s$), based on ADV_NONCONN_IND (ADV NonConn) for non-connectable advertising events and SCAN_REQ (REQ) reception for scannable undirected advertising events (standard and interrupted versions) with and without backoff, as the number of advertisers increases. Comparison between ideal (**a**), type 1 (**b**) and type 2 (**c**) scanning devices, for $T_{advIND}=T_{scanREQ}=$ 176 μs, $T_{scanRSP}=$ 152 μs with $T_{advInterval}=$ 100 ms and $T_{advDelayMax}=$ 10 ms.

Figure 11, connected with the analysis performed in Figure 9, shows the probability of not detecting all the advertising devices and aware of being detected by the scanner (in scannable undirected advertising schemes) when $D_{TH}$ is set to 5 s. Note that, when only ADV_NONCONN_IND and type 1 real devices are considered, the probability of not detecting all the advertisers when $N_{BLE}=200$ is around 0.1%, whereas in the interrupted version all devices are detected when ideal, types 1 and 2 scanning devices are considered. On the other hand, if ADV_SCAN_IND is used as the detection indicator in the interrupted version of the scannable undirected advertising event, the probability that all the devices are detected is 100%.

**Figure 12:**
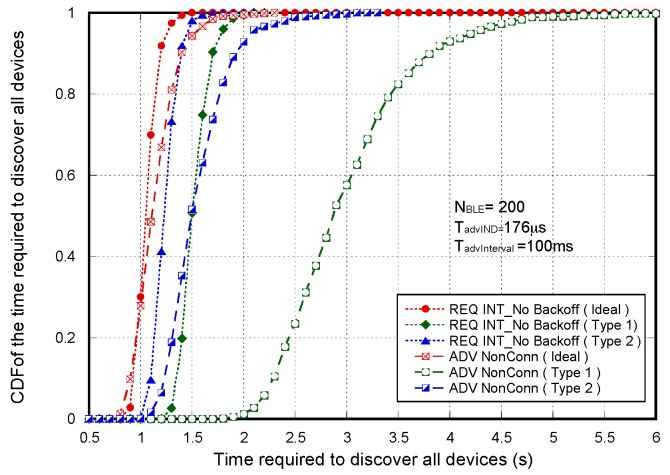
CDF of the time required to discover all the devices when there are *N_BLE_* = 200 BLE advertisers for *T_advIND_ = T_scanREQ_* 176 μs, *T_scanRSP_* = 152 μs with *T_advInterval_ = * 100 ms and *T_advDelayMmax_* = 10 ms. Comparison between the interrupted version of scannable undirected advertising events (based on SCAN_REQ reception) and non-connectable advertising events (based on ADV_NONCONN_IND reception).

In order to better illustrate the analysis and the differences for the two more attractive options (the interrupted version of the scannable undirected advertising events and the non-connectable advertising scheme), Figure 12 depicts the cumulative density function (CDF) of the time required to discover all the devices when *N_BLE_* = 200 advertisers are considered. We can see that the variance is higher when the non-connectable advertising scheme is considered. In fact, as a more restrictive value for *D_TH_* is considered, the higher the advantages of the interrupted version are. For instance, if *D_TH_* is set to 2 s, none of devices are discovered when a type 1 scanning device is considered in a non-connectable advertising scheme, compared to 90% if a type 2 scanning device is used. For its part, the interrupted version guarantees that all the devices are discovered, regardless of whether ideal, type 1 or type 2 scanning devices are considered.

**Figure 13:**
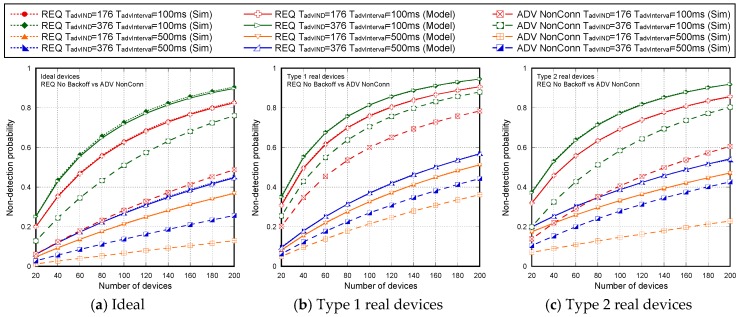
SCAN_REQ (REQ) and ADV_NONCONN_IND (ADV NonConn) non-detection probabilities for scannable undirected (without backoff) and non-connectable advertising events, as the number of advertisers increases. Comparison between the mathematical model and the simulation for ideal (**a**), type 1 (**b**) and type 2 (**c**) scanning devices, for several *T_advIND_* (*T_advIND_* = 176 μs and *T_advIND_* = 376 μs) and *T_advInterval_* values *T_advInterval_* = 100 ms and *T_advInterval_* = 500 ms).

Figure 13 extends the comparison performed in Figure 8 for several *T_advIND_* values (176 µs and 376 µs) and for different *T_advInterval_* (100 ms and 500 ms). 

In contrast to Figure 8, in Figure 13, we focus the analysis on implementations without backoff and non-connectable advertising events. In this case, results are only shown for SCAN_REQ and ADV_NONCONN_IND non-detection probabilities in continuous event configurations. Figure 13a–c shows that mathematical results for SCAN_REQ practically match with the simulation curves when no backoff implementation is considered. Differences between actual devices are significant.

**Figure 14:**
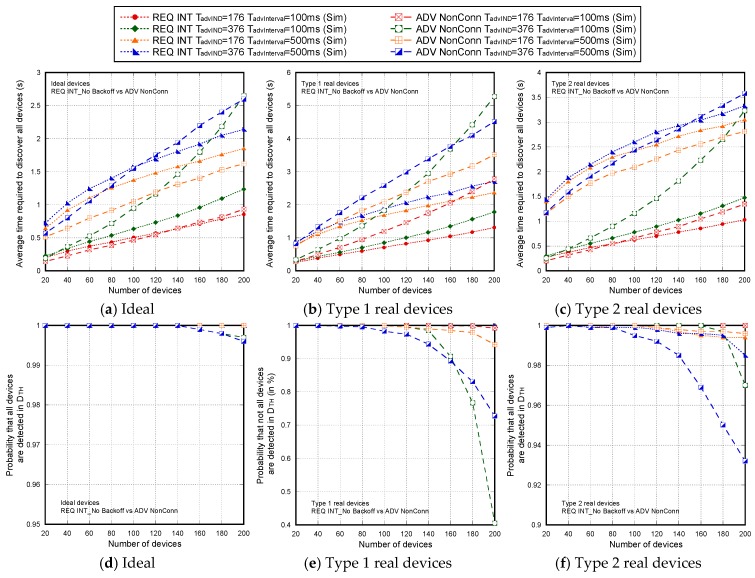
Average time required to discover all devices (**a**–**c**) and probability that all devices are detected in *D_TH_* =5s (**d**–**f**), based on ADV_NONCONN_IND (ADV NonConn) for non-connectable advertising events and SCAN_REQ (REQ) reception for scannable undirected advertising events (interrupted version) without backoff, as the number of advertisers increases. Comparison between ideal (**a**,**d**), type 1 (**b**,**e**) and type 2 (**c**,**f**) scanning devices, for several *T_advIND_* (*T_advIND_* = 176 μs and *T_advIND_* = 376 μs) and *T_advInterval_* values (*T_advInterval_* = 100 ms and *T_advInterval_* = 500 ms).

Connected with Figure 13, Figure 14 shows the average time required to discover all the devices in seconds (Figure 14a–c) and the probability of detecting all the devices in *D_TH_* =5s (Figure 14d–f) for the interrupted version of scannable undirected (INT) and non-connectable advertising scheme (NonConn). Note that upper bounds for the average delay can be obtained (they result in staircase functions) but they are not included in order to facilitate visualization of the comparison. 

Firstly, we see that the differences between actual devices and ideal implementation are significant. On the other hand, the type 2 scanning device offers a better performance than type 1 when the non-connectable option is considered.

**Figure 15:**
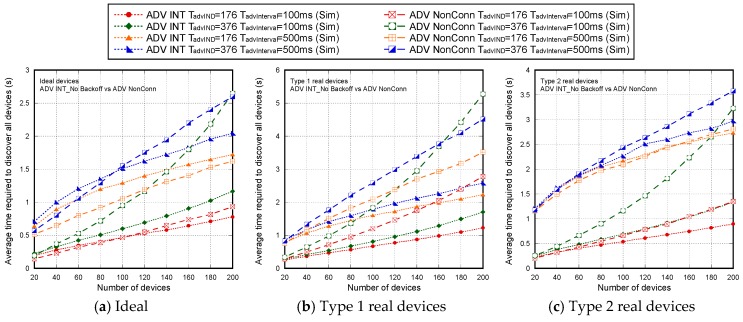
Average time required to discover all devices (**a**–**c**) based on ADV_NONCONN_IND (ADV NonConn) for non-connectable and ADV_SCAN_IND (ADV INT) for scannable undirected advertising events (interrupted version without backoff), as the number of advertisers increases. Comparison between ideal (**a**), type 1 (**b**) and type 2 (**c**) scanning devices, for several *T_advIND_* (*T_advIND_* = 176 μs and *T_advIND_* = 376 μs) and *T_advInterval_* values (*T_advInterval_* = 100 ms and *T_advInterval_* = 500 ms).

Nevertheless, if we focus on the interrupted version of scannable undirected advertising events, we see that the best results are obtained when *T_advInterval_* = 100 ms both for types 1 and 2 devices, although the general conclusions obtained for *T_advInterval_* = 500 ms differ from *T_advInterval_* = 100 ms, no matter the *T_advIND_* values. As we concluded above, type 2 scanning devices offer the best results when *T_advInterval_* = 100 ms. However, real type 1 devices are clearly a better option when *T_advInterval_* = 500 ms. In this case, they not only provide a low average time required to discover all the devices (based on SCAN_REQ reception), but also ensure that the probability that all the devices are detected within *D_TH_* = 5 s is 1. Note that for *T_advInterval_* = 500 ms, ADV_NONCONN_IND and SCAN_REQ non-detection probabilities for type 2 scanners are lower than for type 1 when the number of advertisers is high, but differences are not very significant. On the other hand, as long as the number of devices decreases, the non-detection probabilities are more significant than in type 1 devices due to *scanning gaps*. Thus, if we consider a scenario of, for example, *N_BLE_*, in the first *T_advInterval_* + *T_DelayMax_* interval a higher number of devices are discovered by a type 2 scanner, but as the number of devices remaining to be discovered is reduced, the time required to discover them is higher than when type 1 is considered. At the end, the time required by type 2 is higher. Finally, when comparing the non-connectable advertising option and the adapted version of the scannable undirected advertising option, the latter clearly outperforms the non-connectable advertising option for ideal and type 1 devices for all parameter settings. In fact, the probability that all devices are detected in *D_TH_* = 5 s is significantly lower in the non-connectable option. Concerning type 2, the results are clearly better for *T_advInterval_* = 100 ms and *T_advInterval_* = 500 ms with *T_advIND_* = 376 μs. However, for *T_advInterval_* = 500 ms and *T_advIND_* = 176 μs, the results are not so evident. If SCAN_RSP reception is used as a reference, non-connectable advertising events seem to be more attractive. Nevertheless, in a more fair comparison based on ADV_SCAN_IND reception (see Figure 15), we prove that the performance of type 2 is similar to non-connectable in terms of average delay *T_advInterval_* = 500 ms and *T_advIND_* = 176 μs. On the other hand, it is better in terms of the probability that all devices are detected in *D_TH_* = 5 s (100% of devices are detected). Finally, the comparison of all the schemes based on a fairer discovery indicator (the ADV_SCAN_IND and ADV_NONCONN_IND reception for both scannable undirected and non-connectable advertising events), illustrated in Figure 15, emphasizes the advantages of the proposed method.

In general, we can conclude that effects of parameter settings over different chipset implementations need to be considered. However, it seems clear that the interrupted version of scannable undirected advertising events outperforms results obtained with non-connectable advertising events. On the other hand, the differences between the actual implementations are quite significant and need to be taken into account. Type 1 scanning devices permit the discovery of at least 200 devices in a short period of time, even considering *T_advInterval_* of up to 500 ms and the highest size of ADV_SCAN_IND PDUs. However, the best results are obtained when *T_advInterval_* = 500 ms, both for types 1 and 2 scanning devices. The implementation of the backoff process may severely and unnecessarily degrade the discovery capacities. Thus, it needs to be carefully designed or even deactivated in the intended scenarios. In this sense, although both techniques proposed in [14,15] and mentioned in Section 2.2 would probably work better than the one initially proposed by the standard, in that the advertiser would take less time to receive a SCAN_REQ PDU and, therefore, to find out that it has been discovered, in our scenario, where there is only one scanner, these backoff algorithms would still provide worse performance than the use of no backoff algorithm. In any case, an exhaustive analysis of these algorithms is outside the scope of this article, in which our objective is to demonstrate the improvement when implementing the present proposal compared to previous ones, even considering that a backoff algorithm will diminish the attainable benefits because the advertiser is going to take longer to find out that it has been discovered. Any backoff algorithm that reduces this time, as is the case of those proposed in [14,15], will improve the performance of our proposal with respect to the results obtained with the backoff algorithm initially suggested by the standard.

## 6. Conclusions

A novel proposal for the discovery procedure based on an adapted version of scannable undirected advertising events has been presented. Taking advantage of the new HCI LE Scan REQ Received event introduced in version 5.0, the advertisers can now be disabled temporarily when they are discovered. This new proposal improves the discovery times and the probability of discovery; allows the system to work correctly for a greater number of devices being discovered; provides realistic results, as they derive from a very accurate characterization of the real behavior of the commercial devices; is easily implementable on devices by properly scheduling temporary interruption of the advertising process upon successful reception of an ADV_REQ PDU; and reduces the energy consumption of devices by eliminating unnecessary transmissions of ADV_SCAN_IND PDUs. This results in lower interference, additional energy saving, and device discovery latency reduction, outperforming the results obtained with non-connectable and non-scannable undirected advertising events, previously analyzed in several works. The work takes into account the real behavior of the devices, because real measurements show that, unexpectedly, during scanning and reception, the scanners present several blind times, which reduce the detection capabilities of the system. Differences between the actual implementations are quite significant, and need to be taken into account. All the analyzed chipsets present blind periods that are predictable and are related to the packet decoding process and to particularities of the MAC state implementation. These impairments are not usually considered in the rest of the literature. Nevertheless, this work shows that they should not be omitted, due to their considerable impact on the discovery performance. In this sense, with respect to the representability of the results obtained, it must be taken into account that the advertiser has been considered capable of stopping the process of sending ADV_SCAN_IND PDUs after the transmission of the SCAN_RSP PDU. In practice, it would be necessary to characterize the time that elapses between the advertiser’s receipt of the SCAN_REQ, which allows it to know that it has been discovered, and its effective interruption of the sending of ADV_SCAN_IND PDUs, since there may be latencies caused by the actual implementation of the devices. As there are currently no commercial devices that carry out this characterization, it has not been possible to introduce these times into the model. However, it is expected that the response time will be fast, and will be able to be performed before the next advertising event begins.

In addition, the experimental measurements and simulations show the effects of the backoff algorithm proposed in the specifications due to reception errors or interference. This fact is of vital importance, because, initially, the backoff algorithm was designed to avoid collisions in scenarios with two or more scanners. Nevertheless, the presented results show that the backoff algorithm is also activated when the SCAN_REQ or SCAN_RSP are not received due to errors forced by other packets from other BLE devices in high-density networks, and not only when there is a simultaneous transmission of two SCAN_REQs by several devices in active scanning mode. The backoff mechanism is not really required in a scenario with only one scanner, but its mandatory implementation unnecessarily degrades the discovering capacity. The implementation of the backoff algorithm may be totally different between manufacturers and, in fact, some of the real scanning devices evaluated experimentally in this work do not implement it. Given that this is a challenging issue, it needs to be further studied. In addition to an exhaustive evaluation for different parameter sets and variable numbers of devices, using simulations and real measurements, this work also presents a mathematical model that coincides with the Bluetooth specifications and includes the different singularities of the analyzed chipsets. This model allows the results for any value of the parameters present in the specifications, and for any number of simultaneous advertisers, to be obtained.

## Figures and Tables

**Table 1:** Parameters included in the mathematical model.

Parameter	Description
$T_{advIND}$	Transmission time of the advertising PDU (ADV_SCAN_IND PDU)
$T_{scanREQ}$	Transmission time of the scan request PDU (SCAN_REQ PDU)
$T_{scanRSP}$	Transmission time of the scan response PDU (SCAN_RSP PDU)
$T_{scanRSP}^{Max}$	Maximum transmission time of the scan response (connected with the maximum allowed size of the SCAN_RSP PDU) (376 µs)
$T_{sync}$	Synchronization time
$T_{IFS}$	Inter Frame Space (150 µs)
$T_{sigproc}^{Max}$	Maximum expected time needed for completion of the signalling after succesful detection of the ADV_SCAN_IND ($T_{sigproc}^{Max}=T_{IFS}+T_{scanREQ}+T_{IFS}+T_{scanRSP}^{Max}$)
$T_{advInterval}$	Fixed advertising interval
$T_{advDelayMax}$	Maximum value of the random delay (standard: 10 ms)
$\tau_{advDelay}$	Random delay between advertisements. Uniform [0, *T_advDelayMax_*]
$T_{advEvent}$	Advertising event interval: $T_{advInterval}+\tau_{advDelay}$
$T_{scanInterval}$	Scan Interval
$T_{scanWindow}$	Scan Window
$T_{f}{}_{qChgGap}$	Gap due to change of scanning frequency (types 1 and 2 scanners)
$T_{interFqChgGap}$	Duration of scattered gaps inside the scan interval (type 2 scanner)
$T_{gapInt1}$/$T_{gapInt2}$	Time intervals between scattered gaps inside the scan interval (type 2 scanner)
$T_{minDecodGap}$/$T_{maxDecodGap}$	Minimum and maximum values of processing gap after successful ADV_SCAN_IND or SCAN_RSP PDU detection
$T_{minErrDecodGap}$/$T_{maxErrDecodGap}$	Minimum and maximim values of processing gap after erroneous ADV_SCAN_IND or SCAN_RSP PDU detection
$\tau_{decodGap}$	Processing gap after successful ADV_SCAN_IND PDU or SCAN_RSP PDU detection. Uniform [$T_{minDecodGap}$,$T_{maxDecodGap}$]
$\tau_{errDecodGap}$	Processing gap after erroneus ADV_SCAN_IND PDU or SCAN_RSP PDU detection. Uniform [$T_{minErrDecodGap}$,$T_{maxErrDecodGap}$]
$T_{scanGap}$	Sum of durations of all the gaps occurred on the scan window
$N_{interFqChgGap}^{scanWindow}$	Number of scattered gaps inside the $T_{scanWindow}$
$N_{ngdev}$	Number of neighbor advertising devices
$N_{BLE}$	Total number of advertising devices which are in the coverage area of the scanning device and can potentially collide

**Table 2:** Variables included in the mathematical model (in a scenario with $N_{BLE}$ advertisers).

Variable	Description
$P_{scanGap}^{pattern}$	Probability that a *periodic scanning gap* occurs
$R_{scanGap}^{pattern}$	Rate of periodic scanning
$P_{patternscanGap}^{MAXsigproc}$	Probability of having a *periodic gap* within $T_{advIND}+T_{sigproc}^{MAX}$
$\bar{N_{detAdvIND}}$	Mean number of neighbor devices whose ADV_SCAN_IND are detected within $T_{advInterval}+\bar{\tau_{advDelay}}$
$P_{NDAdvIND}^{col}$	Non-detection probability (P_ND_) of an ADV_SCAN_IND due to collision with another ADV_SCAN_IND
$P_{NDScanREQ}^{col}$	* P_ND_ of a transmitted SCAN_REQ due to collision with an ADV_SCAN_IND
$P_{NDScanRSP}^{col}$	* P_ND_ of a transmitted SCAN_RSP due to collision with an ADV_SCAN_IND
$\bar{T_{sigproc}}$	Mean time the scanner is involved in a signaling processing period within a $T_{advInterval}+\bar{\tau_{advDelay}}$ interval
$P_{NDAdvIND}^{sigproc}$	* P_ND_ of an ADV_SCAN_IND because the scanner is involved in a *signaling processing period*
$\bar{T_{decodGap}}$	Mean time the scanner is involved in *decoding gaps* within a $T_{advInterval}+\bar{\tau_{advDelay}}$ interval
$P_{NDAdvIND}^{decodGap}$	* P_ND_ of an ADV_SCAN_IND because the scanner is involved in a *decoding gap*
$P_{NDAdvIND}^{scanGap}$	* P_ND_ of an ADV_SCAN_IND due to *periodic scanning gaps*
$P_{NDAdvIND}^{gap}$	* P_ND_ of an ADV_SCAN_IND due to *scanning gaps (periodic scanning and decoding gaps)*
$P_{NDAdvIND}^{sigproc+gap}$	* P_ND_ of an ADV_SCAN_IND due to *scanning gaps* and *signaling processing period*
$P_{NDAdvIND}^{}$	Overall * P_ND_ of a transmitted ADV_SCAN_IND
$P_{NDScanREQ}^{}$	Overall * P_ND_ of a transmitted SCAN_REQ
$P_{NdScanRSP}^{}$	Overall * P_ND_ of a transmitted SCAN_RSP
$P_{NDAdvIND}^{+BLER}$	Overall * P_ND_ of a transmitted ADV_SCAN_IND including channel errors (BLER)
$P_{NDScanREQ}^{col+BLER}$	Overall * P_ND_ of a transmitted SCAN_REQ including channel errors (BLER)
$P_{NDScanRSP}^{col+BLER}$	Overall * P_ND_ of a transmitted SCAN RSP including channel errors (BLER)
$P_{allDet}^{advIND}$	Probability of discover of all devices (based on ADV_SCAN_IND) within a D_TH_ interval.
$P_{allDet}^{scanREQ}$	Probability of discovering all devices (based on SCAN_REQ) within a D_TH_ interval.
$\bar{D_{allDet}^{advIND}}$	Average time required to discover all devices based on ADV_SCAN_IND.
$\bar{D_{allDet}^{scanREQ}}$	Average time required to discover all devices based on SCAN_REQ
$\bar{N_{req}^{xpdu}}$	Average number of pdu (xpdu = ADV or SCAN_REQ) transmissions required before detection of a device
$\bar{D_{\det ect}^{xpdu}}$	Average detection delay of a pdu (xpdu = advIND or scanREQ) transmitted by a device
$\bar{t_{interDetect}^{xpdu}}$	Average time between two consecutive detections of a device (based on xpdu = advIND or scanREQ)
$\bar{N_{detect}^{xpdu}}$	Average number of detections of an advertiser BLE within a window of opportunity (based on xpdu = advIND or scanREQ)
$D_{TH}$	Time threshold for detection
$T_{covWindow}$	Coverage time interval or dwell time

* P_ND_: non-detection probability.

**Table 3:** Parameters used in the evaluation.

General Parameters		Real Scanner Service Parameters		
Parameter	Values	Parameter	Values	
			Type 1	Type 2
$T_{advIND}$	176 μs, 376 μs	$T_{fqChgGap}$	1.1 ms	16.04 ms
$T_{advInterval}$	100 ms, 500 ms	$T_{interFqChgGap}$	-	274 μs
$T_{advDelayMax}$	10 ms	$T_{gapInt}{}_{1}$	-	16.82 ms
$T_{scanInterval}$/$T_{scanWindow}$	500 ms/500 ms	$T_{gapInt}{}_{2}$	-	4.3 ms
$T_{covWindow}$/$D_{TH}$	20 s/5 s	$T_{minDecodGap}$/$T_{minErrDecodGap}$	350 ms/350 ms	194 μs/144 μs
$N_{BLE}$	2–200	$T_{maxDecodGap}$/$T_{maxErrDecodGap}$	1.6 ms/1.6 ms	194 μs/144 μs
